# Alteration of gene expression and protein solubility of the PI 5-phosphatase SHIP2 are correlated with Alzheimer’s disease pathology progression

**DOI:** 10.1007/s00401-024-02745-7

**Published:** 2024-06-04

**Authors:** Kunie Ando, Fahri Küçükali, Emilie Doeraene, Siranjeevi Nagaraj, Eugenia Maria Antonelli, May Thazin Htut, Zehra Yilmaz, Andreea-Claudia Kosa, Lidia Lopez-Guitierrez, Carolina Quintanilla-Sánchez, Emmanuel Aydin, Ana Raquel Ramos, Salwa Mansour, Sabrina Turbant, Stéphane Schurmans, Kristel Sleegers, Christophe Erneux, Jean-Pierre Brion, Karelle Leroy, Brain Bank NeuroCEB Neuropathology Network

**Affiliations:** 1 Alzheimer and Other Tauopathies Research Group, ULB Neuroscience Institute (UNI), ULB Center for Diabetes Research (UCDR), Faculty of Medicine, Université Libre de Bruxelles, 808 Route de Lennik, Bldg GE, 1070 Brussels, Belgium; 2 Complex Genetics of Alzheimer’s Disease Group, VIB Center for Molecular Neurology, Antwerp, Belgium; 3 Department of Biomedical Sciences, University of Antwerp, Antwerp, Belgium; 4 Laboratory of Histology, Neuroanatomy and Neuropathology, Faculty of Medicine, Université Libre de Bruxelles, ULB Neuroscience Institute (UNI), 808 Route de Lennik, 1070 Brussels, Belgium; 5 Institute of Interdisciplinary Research in Molecular Human Biology (IRIBHM), Université Libre de Bruxelles, 808 Route de Lennik, 1070 Brussels, Belgium; 6 Biobanque Neuro-CEB, Hôpital de la Pitié-Salpétrière, Paris, France; 7 Plateforme de Ressources Biologiques (PRB), Hôpital de La Pitié-Salpêtrière, AP-HP, Paris, France; 8 Laboratory of Functional Genetics, GIGA Research Centre, University of Liège, Liège, Belgium

**Keywords:** Alzheimer’s disease, SHIP2, INPPL1, EGFR, Tau, Amyloid ß, GWAS, CSP pTau

## Abstract

A recent large genome-wide association study has identified *EGFR* (encoding the epidermal growth factor EGFR) as a new genetic risk factor for late-onset AD. SHIP2, encoded by *INPPL1*, is taking part in the signalling and interactome of several growth factor receptors, such as the EGFR. While *INPPL1* has been identified as one of the most significant genes whose RNA expression correlates with cognitive decline, the potential alteration of SHIP2 expression and localization during the progression of AD remains largely unknown. Here we report that gene expression of both *EGFR* and *INPPL1* was upregulated in AD brains. SHIP2 immunoreactivity was predominantly detected in plaque-associated astrocytes and dystrophic neurites and its increase was correlated with amyloid load in the brain of human AD and of 5xFAD transgenic mouse model of AD. While mRNA of *INPPL1* was increased in AD, SHIP2 protein undergoes a significant solubility change being depleted from the soluble fraction of AD brain homogenates and co-enriched with EGFR in the insoluble fraction. Using FRET-based flow cytometry biosensor assay for tau-tau interaction, overexpression of SHIP2 significantly increased the FRET signal while siRNA-mediated downexpression of SHIP2 significantly decreased FRET signal. Genetic association analyses suggest that some variants in *INPPL1* locus are associated with the level of CSF pTau. Our data support the hypothesis that SHIP2 is an intermediate key player of EGFR and AD pathology linking amyloid and tau pathologies in human AD.

**Supplementary Information:**

The online version contains supplementary material available at 10.1007/s00401-024-02745-7.

## Introduction

Alzheimer’s disease (AD), the most common form of dementia, is characterized by two main neuropathological lesions: amyloid plaques and neurofibrillary tangles (NFTs). Amyloid plaques are constituted of amyloid β (Aβ) peptides [39] derived from successive cleavages of the amyloid precursor protein (APP). NFTs are composed of hyperphosphorylated and aggregated PHF-tau (paired-helical filament) [15]. NFTs are also found in other neurodegenerative diseases so called “tauopathies” such as familial forms of frontotemporal lobar degeneration with tau-immunoreactive inclusions (FTD-tau), corticobasal degeneration (CBD), progressive supranuclear palsy (PSP), argyrophilic grain disease (AGD), Pick disease (PiD) and others [17, 88]. AD is closely linked to dysfunction of membrane sorting and phosphoinositide (PI) homeostasis [64]. Membrane dynamics are tightly regulated by membrane lipids such as PIs [27, 80]. In the affected brain areas of AD brains, enlargement of endosomes precedes the formation of amyloid plaques [19].

Recent network-based approach studies have revealed that upregulation of SHIP2 (SH2 domain-containing Inositol 5-Phosphatase 2) transcripts encoded by *INPPL1* (Inositol Polyphosphate Phosphatase Like 1) is significantly associated with cognitive decline and neuropathological lesions in human AD and aging brains [65] and that *INPPL1* is one of the most significant AD-associated genes that can be potential diagnostic biomarkers and therapeutic targets for AD [24]. SHIP2 is one of the ten PI 5-phosphatases identified in the human genome [72]. SHIP2 regulates the levels of the critical second messenger PI(3,4,5)P3 by essentially dephosphorylating PI(3,4,5)P3 at the 5-position to produce PI(3,4)P2 [37, 71]. SHIP2 is ubiquitously expressed and is implicated in several human diseases such as metabolic syndrome, diabetes [23], breast cancer [35] and Opsismodysplasia [33]. Since PI content and regulation are prominently altered in AD brains [80], SHIP2 inhibitors are under active scrutiny as novel therapeutic targets for AD [58]. Lentivirus-mediated downregulation of SHIP2 in astrocyte culture significantly reduced Aβ production [65]. Other independent studies have consistently suggested the involvement of SHIP2 in AD, showing that SHIP2 mediates amyloid-toxicity via either actin-cytoskeleton reorganization [53] or tau hyperphosphorylation [48].

Of note, SHIP2 is a part of EGFR (epidermal growth factor receptor) interactome [60, 70] and EGF stimulation triggers TOM1L1 (Target Of Myb1 Like 1 Membrane Trafficking Protein) and Fyn-dependent recruitment of SHIP2 to clathrin-coated pits [18]. Intriguingly, a recent large genome-wide association study (GWAS) has identified *EGFR* as one of the most significant novel risk genes for late onset Alzheimer’s disease (LOAD) [13] raising the question of the possible participation of SHIP2 in EGFR function/turnover in AD. Nonetheless, the gene expression levels of *EGFR* and *INPPL1*, the protein levels of EGFR and SHIP2 and the potential association of SHIP2 with AD lesions in post-mortem AD brain tissues remain largely unknown.

Here, we report a significant increase in gene expression of *EGFR* and *INPPL1* and protein level of EGFR and SHIP2 in the highly insoluble fraction enriched with pathological protein aggregates in AD brains. In the presence of amyloid pathology, there was a significant increase of SHIP2-immunoreactivity observed around amyloid plaques in AD brains and in 5xFAD transgenic mouse brains bearing amyloid pathology [67]. Overexpression of SHIP2 led to increased FRET signal in HEK tau biosensor model [43]. Finally, genetic analysis suggested that some *INPPL1* genetic variants are associated with CSF phosphorylated tau (pTau) levels. Altogether, these data suggest that SHIP2 plays a role in the link between amyloid and tau pathologies in AD.

## Materials and methods

### Antibodies

Mouse monoclonal anti-SHIP2 (3E6) antibody was purchased from Novus (H00003636-M01), Cambridge, UK. Rabbit polyclonal anti-SHIP2 X5A and X4 antibodies were generated in rabbits with human C-terminal peptide of SHIP2 (C-terminal peptide of 1243–1258 amino acids, namely DPAHKRLLLDTLQLSK) [66]. Mouse monoclonal anti-actin (A-5441), rabbit polyclonal anti-Aß42 (AB5078P), rabbit polyclonal anti-GAPDH (G-4644) antibodies were purchased from Sigma-Merck. Mouse monoclonal anti-Aß (6E10) was purchased from Covance. Mouse monoclonal anti-pTau (Ser202 and T205) AT8 antibody was purchased from ThermoFisher. Mouse monoclonal anti-pTau (Ser396 and Ser404) PHF1 antibody was provided by Dr. Peter Davies (Albert Einstein College of Medicine, NY) [68]. Rabbit polyclonal anti-tau (A0024) and rabbit polyclonal anti-myelin basic protein (N1546) antibodies were purchased from Dako. Rabbit polyclonal anti-AQP1 antibody (A-2219) was purchased from Millipore. Goat polyclonal anti-Iba1 antibody (ab5036) was purchased from Abcam.

### Human brain tissues

Frozen samples from the temporal superior T1 isocortex were obtained from AD and age-matched non-demented control subjects. Control cases were non-demented individuals who died without known neurological disorders. AD cases were diagnosed according to the National Institute of Aging and Reagan Institute Criteria [12] and scored by neuropathological staging for tau and amyloid pathologies [14, 83]. AD cases including two familial Alzheimer’s disease (FAD) cases with *Amyloid Precursor Protein (APP)* or *Presenilin1* (*PSEN1*) mutations and one case of Down syndrome with AD lesions (DS/AD) were all scored as Braak’s stage V or VI (Supplementary Table 1). Paraffin-embedded tissues were also analysed in control, AD and other related neurodegenerative cases as cerebral amyloid angiopathy (CAA), Lewy body disease (LBD), frontotemporal dementia (FTD) with TDP-43 positive inclusions, PSP, CBD, PiD and FTD with abnormal tau (FTD-tau) with mutation of *MAPT* (microtubule associated protein tau). The mean ages and post-mortem delays of control cases and of AD patients were not significantly different. Average age at death was 74.3 ± 10.7 and 72.7 ± 12.4 years for control (*n* = 38) and disease (*n* = 76) cases, respectively (mean ± SD) (*p* = 0.54). Average post-mortem delays were 24.0 ± 14.4 h and 25.1 ± 15.2 h for control and AD cases (mean ± SD) (*p* = 0.59). *APOE* genotype was determined for the cases with an informed consent for genetic study using PCR amplification for genomic DNA and sequencing as described [82]. Non-demented control and AD individuals were enrolled in a brain donation program of the French national network of Brain Bank, GIE NeuroCEB, organized by a consortium of Patients Associations or in Belgian ULB brain bank. For GIE NeuroCEB brain samples, an explicit consent had been signed by the patient or by the next of kin, in the name of the patient. The project was approved by the scientific committee of the Brain Bank. The whole procedure of the Brain Bank has been reviewed and accepted by the Ethical Committee “Comité de Protection des Personnes Paris Ile de France VI” and has been declared to the Ministry of Research and Higher Education as requested by the French law. The cases obtained from ULB brain bank (BB190052) were studied in compliance and following approval of the Ethical Committee of the Medical School of the Université Libre de Bruxelles (ULB).

### Preparation of brain homogenates for biochemical analysis

About 200 mg of frozen T1 isocortex grey matter was homogenized as reported [2, 8] (Supplementary Fig. 1) in 10 volumes of ice-cold RIPA buffer containing 50 mM Tris–HCl pH 7.4, 50 mM NaCl, 1% NP-40, 0.25% sodium deoxycholate, 5 mM EDTA, 1 mM EGTA, complete protease inhibitor cocktail (Merck, 11,697,498,001), 1 mM PMSF (Sigma, P-7626), and phosphatase inhibitor cocktail 2, (Sigma, P-5726) and incubated for 60 min at 4 °C on a rotator. 100 µl of the total homogenate was supplemented with Laemmli sample buffer, sonicated on ice and analysed as the total fraction. The rest of the total homogenates was centrifuged (20,000 × *g* for 20 min at 4 °C) and the supernatant was used as a RIPA-soluble fraction. The RIPA-insoluble pellet was sonicated on ice (10 pulses of 1 s with 1 s interval) in fivefold volume of 8 M urea containing protease and phosphatase inhibitors and incubated for 30 min at room temperature on a rotator. The mixture was centrifuged at 20,000 × *g* at 4 °C for 30 min. The supernatant was used as RIPA-insoluble fraction. For each fraction, protein concentrations were estimated by the Bradford method (Bio-Rad, #5000205) before addition of Laemmli sample buffer. 25 µg of protein was loaded to each well for SDS-PAGE.

### Preparation of human Sarkosyl insoluble PHF-tau fraction

Sarkosyl fractionation of human brain tissue was carried out as previously described [7, 16, 40]. 0.5 g of frozen frontal cortex from control (Braak I, Thal 0) and AD (Braak V-VI, Thal 4) cases was homogenized in 10 volumes of ice-cold PHF-extraction buffer (10 mM Tris–HCl (pH 7.4), 0.8 M NaCl, 1 mM EDTA, 10% sucrose). The homogenate was centrifuged at 15,000 × *g* for 20 min at 4 °C. N-lauroylsarcosine sodium salt (L-5125; Sigma-Aldrich) was added to the supernatant to reach a final concentration of 1% (w/v). The lysate was incubated overnight with a mild agitation at 4 °C followed by an ultracentrifugation at 180,000 × *g* for 30 min at 4 °C. The Sarkosyl soluble supernatant was removed, and the Sarkosyl insoluble pellet was briefly rinsed and re-suspended in 0.25 ml of PBS (pH 7.4) by vigorous pipetting. The protein concentration was determined by Bradford protein assay (Bio-Rad) and adjusted to 1 mg/ml. This Sarkosyl insoluble PHF-tau fraction was aliquoted and kept at -80 °C. Sarkosyl-insoluble fractions were analysed by Western blotting (WB) and transmission electron microscopy as previously described [8, 9].

### Analyses of RNA expressions human data sets

RNA-Seq data of the Mayo Clinic cohort were obtained from the AD Knowledge Portal. Detailed information was described in the previous reports on the sample collection, post-mortem sample data, tissue and RNA preparation, library preparation and sequencing, and sample quality controls [1]. The whole transcriptome data based on RNA-Seq were generated from temporal cortex (TCX) human samples obtained from the Mayo Clinic TCX RNA-Seq dataset (https://www.synapse.org/#!Synapse:syn4650265) [1]. The normalized RNA expression of elderly controls and AD were analysed from datasets according to the criteria of Braak staging [14].

### Western blot (WB)

Tissue lysates were run in 7.5% Tris–Glycine gels and transferred onto nitrocellulose membranes (sc-3724, Santa Cruz Biotechnology). The nitrocellulose membranes were blocked in 10% (w/v) semi fat dry milk in TBS (Tris–HCl 0.01 M, NaCl 0.15 M, pH 7.4) for 1 h at room temperature and were incubated with primary antibodies overnight followed by rinses and an incubation with anti-rabbit (#7074, Cell Signaling Technology) or anti-mouse (A-6782, Sigma) immunoglobulin antibodies conjugated to horseradish peroxidase. After several rinses, the membranes were incubated with SuperSignal West Pico PLUS Chemiluminescent Substrate (Pierce) and were exposed to a DARQ-7 CCD cooled camera (Vilber-Lourmat) in a SOLO 4S WL system. Levels of optical density (OD) of protein signals were estimated by densitometric analysis using the NIH ImageJ program. OD of GAPDH signal was used to normalize protein loading.

### Immunohistochemistry

After formaldehyde fixation (10% buffered formalin), brain tissues were paraffin embedded and sliced in 7 µm thick sections. Staining by 3,3ʹ-diaminobenzidine (DAB) was performed as previously described [2]. For 3E6 staining, deparaffinized and rehydrated tissue sections were heated in citrate buffer for 20 min. For 6E10 staining, the sections were incubated in 100% formic acid for 15 min [55]. The sections were incubated in H_2_O_2_ to inhibit endogenous peroxidase, rinsed in water and then incubated with a blocking solution containing 10% normal goat serum (NGS) in TBS. Sections were then incubated overnight with the primary antibody (1/200 for 3E6, 1/1000 for 6E10 and 1/500 for AT8). After several rinses with TBS, the sections were incubated with secondary antibody, biotin-conjugated goat anti-mouse IgG (Vector, BA-9200) at 1/100 in 1% NGS. After several rinses with TBS and incubation with ABC-HRP (VEC. PK-6100, Vectastain), the immunolabelling was visualized using diaminobenzidine (DAB) as chromogen (K346811, Agilent Technologies). After dehydrating and mounting, the sections were observed with Leica DM500 microscope equipped with ICC50 camera. For quantitative analysis of SHIP2 (3E6), Aβ (6E10) and pTau (AT8) staining, hippocampal CA1-2 pyramidal layer was analysed on images taken with 40X objectives by thresholding analyses using NIH ImageJ as previously reported [85].

Double immunofluorescence labelling was performed as previously reported [3]. Mouse monoclonal antibodies were used at 1/100 and detected with a donkey anti-mouse antibody conjugated with Alexa488 (A21206, Invitrogen). Rabbit or goat polyclonal antibodies were used at 1/100 and detected using a biotin-labelled secondary antibodies (A16027 for rabbit IgG and A16009 for goat IgG, Invitrogen) followed by incubation with Streptavidin-Alexa594 (S11227, Invitrogen). Slides were counterstained with DAPI and mounted with Glycergel (Dako). Immunofluorescence labelling was observed with an Axiovert 200 M microscope equipped with an ApoTome system (Zeiss).

### Mouse lines

The 5xFAD double transgenic mice co-express and co-inherit the 695 amino acids isoform of the human amyloid precursor protein (APP695) carrying the Swedish, Florida, and London mutations and the human presenilin-1 (PS1) carrying the M146L and L286V mutations (Tg6799 line) [67]. Tg30 mice express a 1N4R human tau isoform mutated at positions G272V and P301S, under Thy1.2 promoter [56, 77]. 5xFAD and Tg30 mice were crossed to generate 5xFAD X Tg30 mouse line [42]. Only heterozygous transgenic mice of 5xFAD, Tg30 and 5xFAD X Tg30 were used for this study. Genotyping was performed by PCR amplification of genomic DNA as reported previously [55]. The mouse model Ship2^Δ/Δ^ is a mouse model expressing catalytically inactive mouse Ship2 protein [29]. Homozygous Ship2^Δ/Δ^ and its wild-type littermates were compared. All the mouse lines were maintained on C57BL/6 J genetic background. All studies on animals were performed in compliance with and after approval of the Ethical committee for the care and use of laboratory animals of the Medical School of the ULB.

### Cell culture

Tau RD P301S FRET Biosensor cells (CRL-3275) were purchased from ATCC. This cell line was derived by transducing HEK293T cells with 2 separate lentivirus constructs encoding tau RD P301S-CFP and tau RD P301S-YFP [43]. The cells were cultured in Dulbecco’s Modified Eagle’s Medium (DMEM) supplemented with 10% foetal bovine serum (FBS), 100 I.U./ml penicillin/streptomycin and 2 mM L-Glutamine (Gibco) in a humidified incubator at 37 °C with 5% CO_2_ as previously described [25].

### Liposome-mediated transduction of Sarkosyl insoluble fraction containing AD-PHF in tau RD P301S FRET Biosensor cells

Tau RD P301S FRET Biosensor cells were plated at a density of 40,000 cells per well in a 6-well plate. 24 h later, at 60% confluency, cells were transduced with plasmid (empty vector or pcDNA3-His human SHIP2) [70]. Transduction complexes were made by combining 1 µg of plasmid, 1 µg of sarkosyl insoluble fraction (AD-PHF or control), 2 µl jetPRIME (Polyplus) reagent with jetPRIME buffer for a total volume of 100 μl per well. Liposome preparations were incubated at room temperature for 10 min before adding to cells cultured in 1 ml complete medium per well. For analysing the reduction of SHIP2, 2 µl of 20 nmol stock of scramble siRNA control (Dharmacon #D-001910-10-50) or siRNA targeting *INPPL1* (Invitrogen #1,299,001) was co-transfected with 1 µg of Sarkosyl insoluble fraction as described above. Cells were incubated with transduction complexes for 48 h before harvesting.

### Fluorescence activated cell sorting (FACS)

For fluorescence activated cell sorting (FACS), the cells in 6-well plates were harvested with 300 µl 0.05% Trypsin–EDTA (Gibco), mixed with 1 ml complete medium and centrifuged at 1000 × *g* for 5 min to make a cell pellet [78]. The collected cells were re-suspended in 500 µl sterile PBS containing 2% FBS and analysed for FRET flow cytometry (BD LSRFortessa™ X-20 Cell Analyzer). The integrated mean fluorescence intensity was normalized to that of empty vector or scramble siRNA control co-transduced with AD-PHF.

### Western blot and immunocytochemistry *of tau* RD P301S FRET Biosensor cells

For WB, the cells were rinsed three times with PBS and then scraped with Laemmli sample buffer and sonicated on ice. The cell lysates were analysed by WB. For immunocytochemistry, the cells on coverslips were rinsed three times with PBS and were fixed for 10 min with 4% paraformaldehyde (PFA) in PBS containing 4% Sucrose. After the fixation, the cells were rinsed in TBS, permeabilized for 5 min with TBS supplemented with 0.3% Triton X-100, blocked with 10% normal goat serum and were immunostained for anti-SHIP2 antibody (3E6) at 1/200 in 1% NGS in TBS overnight at 4 °C. After three rinses, the cells were incubated with biotin-conjugated goat anti-mouse (H + L) (BA-9200, Vector Laboratories) at 1/100 for 30 min. After three rinses in TBS, the cells were incubated with streptavidin-Alexa594 (S11227, Invitrogen) supplemented with DAPI for 30 min at room temperature. After three rinses with TBS, the cells were mounted in Glycergel (Dako).

### Statistical analyses

Numbers of samples were indicated in the figure legends. Statistical analyses were performed using the Prism 9 program (GraphPad Software). Statistical comparisons were performed using unpaired two-tailed Student *t *tests, one-way ANOVA or two-way ANOVA tests as noted in figure legends. Values of *p* < 0.05 were considered significant.

### Genetic association analyses

We investigated the associations between *INPPL1* locus genetic variants and AD and AD-relevant CSF biomarkers in the publicly available summary statistics of large-scale GWAS. We used the European Alzheimer & Dementia Biobank (EADB) AD GWAS stage I meta-analysis results (*n* = 4,87,511, of 39,106 were clinically diagnosed AD cases & 46,828 were proxy cases, and 4,01,577 were controls) for AD risk [13], while the EADB CSF GWAS stage I meta-analysis results were used for CSF Aß42 (*n* = 8074) and pTau (*n* = 7798) biomarker levels. The genetic variants within 1 Mb of *INPPL1* gene coordinates (hg38 chr11:71,224,767–73,239,147) in these GWAS summary statistics were considered for the genetic association analyses. Linkage disequilibrium (LD) R-squared (*r*^*2*^) values between the associated variants were derived from 1000 Genomes (1 KG) project non-Finnish European samples (*n* = 404) [34, 59]. Brain expression quantitative trait loci (eQTL) information was based on *n* = 560 subjects of The Religious Orders Study and Memory and Aging Project (ROSMAP) cohort with dorsolateral prefrontal cortex (DLPFC) RNA-Seq and whole genome sequencing (WGS) datasets available, as previously mapped and described [13]. GWAS-eQTL colocalization analyses were performed using coloc [36]. Finally, the gene-based rare variant association analysis (RVAS) results for *INPPL1* were queried in large-scale analyses for AD risk [44], for AD-relevant biomarker traits [51], and other phenotypic traits [49].

## Results

### Gene expression of EGFR and INPPL1 is upregulated in AD brains and their protein levels are increased in the insoluble fraction of AD brains

**Fig. 1:**
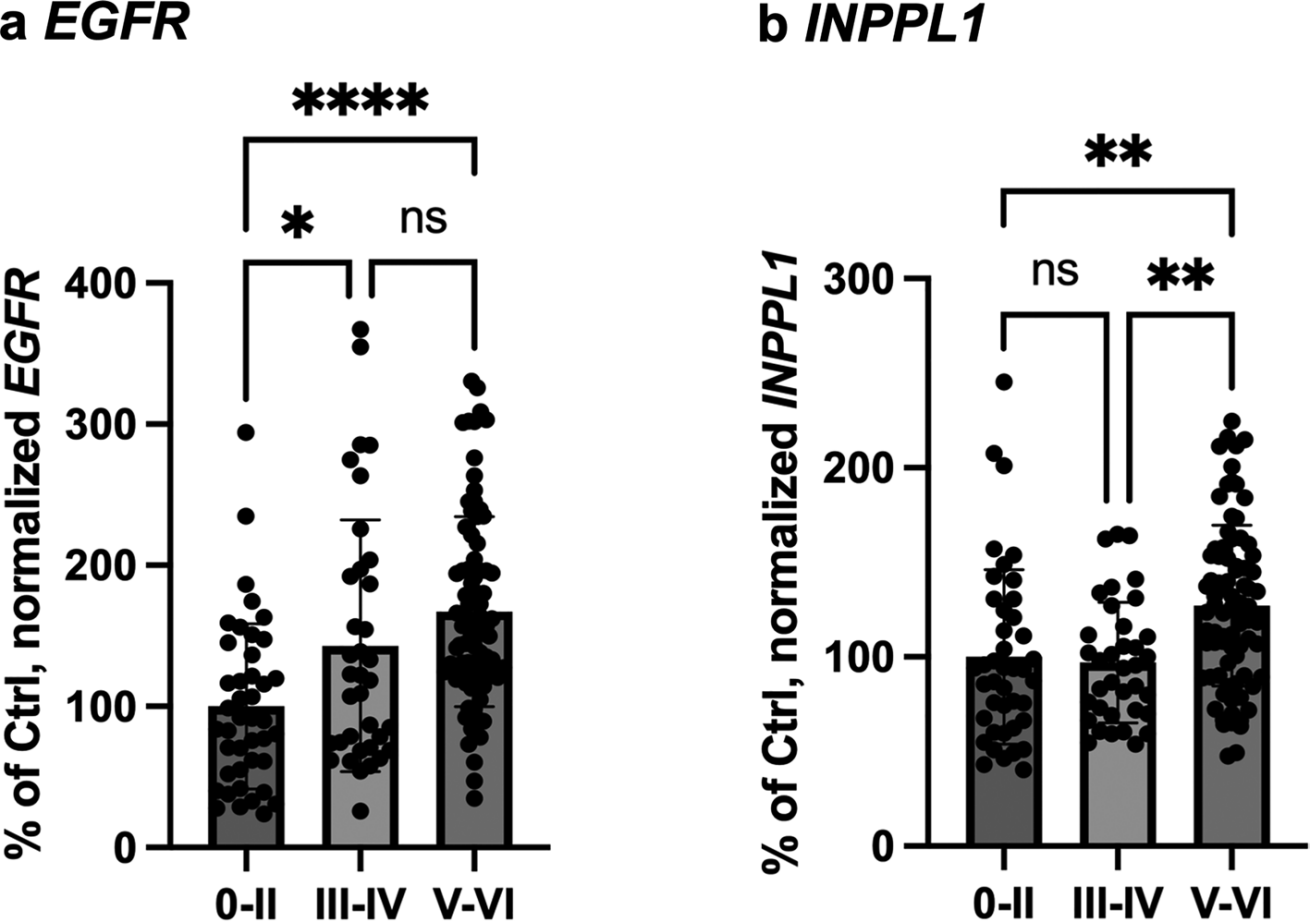
Gene expression of *EGFR* and *INPPL1* is upregulated in AD brains. **a-b** A significant increase of normalized transcripts of *EGFR* and *INPPL1* was observed in AD brains of Mayo clinic TCX cohort (Braak 0-II *n* = 42, Braak III-IV *n* = 36, Braak V-VI *n* = 82). Normalized gene expression of *EGFR* (**a**) and *INPPL1* (**b**) was significantly increased in AD brains. ****p < 0.0001, *p < 0.05 for *EGFR* and **p < 0.01 for *INPPL1* by one-way ANOVA test with a Tukey’s multiple-comparisons post hoc test. 100% was given to the average of normalized mRNA level in the control with Braak 0-II

Since SHIP2 is a part of EGFR interactome [60, 70] and *EGFR* has been recently reported as a new genetic risk factor for LOAD [13], we analysed their gene expression levels in the Mayo clinic TCX cohort. We found that the normalized gene expression of *EGFR* was significantly upregulated in the AD samples of Mayo clinic TCX cohort (Fig. 1a). The gene expression of *INPPL1* was also significantly upregulated in the AD samples with Braak stages V-VI as compared to control samples with Braak stages 0-II (Fig. 1b). These data led us wonder whether EGFR and SHIP2 may be synergistically involved in the development of AD pathologies.

**Fig. 2:**
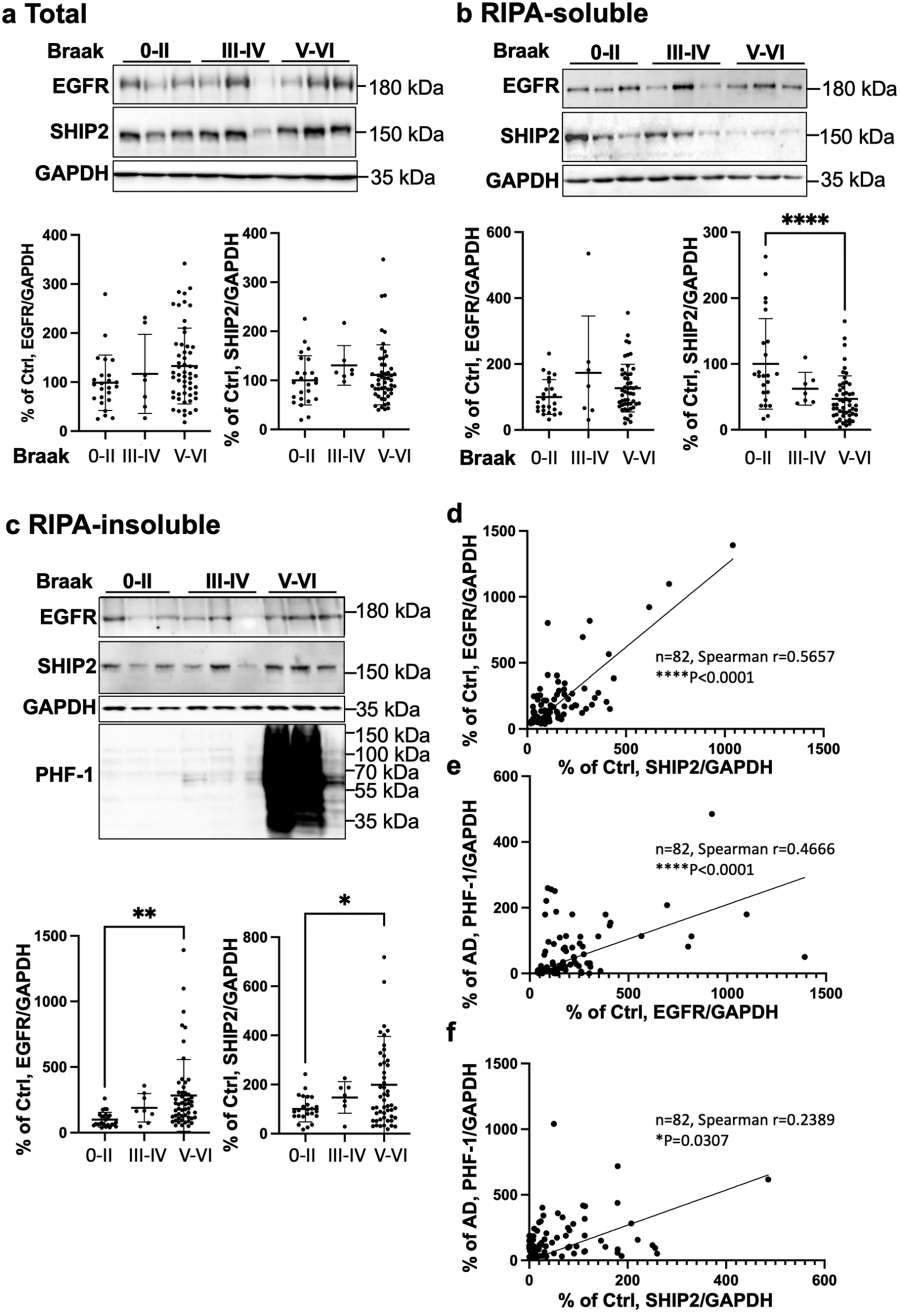
SHIP2 and EGFR proteins are increased and co-enriched in the RIPA-insoluble fraction of AD brains. **a** In the total homogenate of human brain T1 isocortex, normalized EGFR and SHIP2 protein levels were not significantly altered in the T1 isocortex brain lysates of AD compared to those of control non-demented. **b** In the RIPA-soluble fraction of AD brains, SHIP2 protein level was largely reduced. In the same fraction, EGFR protein level was not significantly altered in AD brains compared to control. **c** Both EGFR and SHIP2 protein levels were increased in RIPA-insoluble fraction of AD brains. **d-f** Spearman non-parametric analyses showed significant correlations between EGFR and SHIP2 protein levels (Spearman *r* = 0.5657, ****p < 0.0001) (**d**), between pTau (measured using PHF-1 antibody) and EGFR (Spearman *r* = 0.4666, ****p < 0.0001) (**e**) and pTau and SHIP2 (Spearman *r* = 0.2389, *p = 0.0307) (**f**) in the insoluble fraction. T1 isocortex from control of Braak 0-II (*n* = 24), Braak III-IV (*n* = 7) and AD (*n* = 51) including 2 FAD cases with *APP* or *PSEN1* mutation were analysed. *p < 0.05, **p < 0.01, ***p < 0.001, ****p < 0.0001 by one-way ANOVA test with a Tukey’s multiple-comparisons post hoc test. (**a-c**) or by Spearman non-parametric correlation test (**d-f**). 100% was given to the average of the control non-demented group data normalized to GAPDH

We subsequently analysed EGFR and SHIP2 protein expression by WB in brain lysates of T1 isocortex (Fig. 2). SHIP2 positive bands were detected around 155 kDa in control and AD brains as reported for SHIP2 in previous studies in human and mouse cell models [35, 66]. Normalized SHIP2 protein level with respect to GAPDH was not significantly altered in total fraction of AD brains (Fig. 2a). While SHIP2 is known to be localized in the cytosol, it can be redistributed upon EGF stimulation by associating to membranes via its interaction with a large number of cytoskeletal proteins and/or adaptors [32, 35, 60, 73, 76, 80]. We therefore asked whether the subcellular distribution of SHIP2 could be altered in AD brains. Fractionation analyses suggested changes in SHIP2 partitioning between soluble and insoluble fractions in AD brains (Fig. 2b–c). While SHIP2 was highly detected in the RIPA-soluble fraction of control non-demented brain lysates, SHIP2 was hardly detected in that of the AD brain lysates (Fig. 2b). In contrary, SHIP2 protein level was increased in the RIPA-insoluble fraction of AD brains (Fig. 2c). These data suggest that SHIP2 was predominantly sequestered to the insoluble fraction in AD brains. Interestingly, whereas EGFR protein level was not significantly decreased in the RIPA-soluble fraction of AD brain lysates unlike SHIP2 (Fig. 2b), EGFR was also increased in the RIPA-insoluble fraction of AD brains (Fig. 2c). The normalized levels of EGFR and SHIP2 were significantly correlated in the RIPA-insoluble fraction and were correlated with the progression of AD as measured by pTau level using PHF-1 antibody (Fig. 2d). These biochemical data coincide with the increased gene expression of *EGFR* and *INPPL1* (Fig. 1). Taken together, gene expression of both *EGFR* and *INPPL1* is increased in AD brains and SHIP2 protein undergoes alteration in its apparent protein solubility during the progression of AD: SHIP2 is enriched in the insoluble fraction in AD brains together with its known interactor EGFR.

### SHIP2 immunoreactivity is increased in the presence of amyloid pathology

**Fig. 3:**
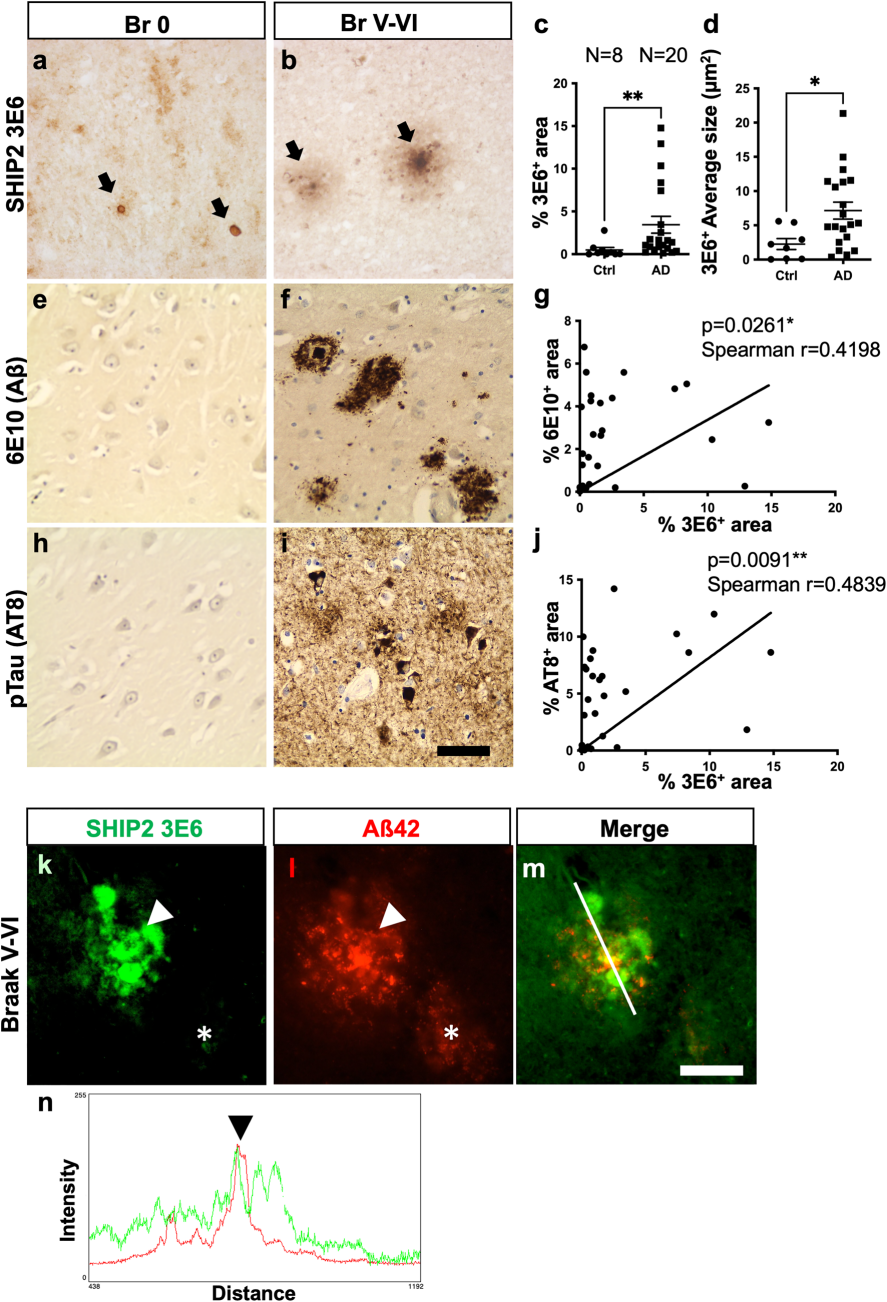
SHIP2 immunoreactive structures are increased in AD brains and partially associated with amyloid plaques. **a-b** Representative images of immunostaining of SHIP2 using 3E6 antibody in CA1-2 hippocampal area of a control (**a**) and an AD (**b**) cases. Black arrows show 3E6-positive structures. **c-d** The average size (**c**) and percentage of the area occupied by SHIP2-immunolabelling (**d**) were significantly increased in the CA1-2 of AD brains compared to controls. **e–f, h-i** Representative images of immunostaining of anti-Aβ 6E10 (**e–f**) and anti-pTau AT8 (**h-i**) antibodies in CA1-2 hippocampal area of a control (**e, h**) and an AD (**f, i**) cases. **g, j** There were significant correlations between SHIP2 positive area and amyloid load (**g**) or tau load (**j**). *p < 0.05 and **p < 0.01 by Mann–Whitney U test (**c, d**) or by Spearman non-parametric correlation analyses (**g, j**). **k-n.** A double immunostaining for SHIP2 (**k**, *green*) and Aß42 (**l**, *red*) suggested that SHIP2 immunoreactivity was partly associated with amyloid plaques. Mature dense-core plaque (arrowhead) showed accumulation of SHIP2-immunolabelled structure while diffuse plaque (asterisk) showed a weak SHIP2 immunoreactivity (**m** *merge*). *Scale bars* 20 μm. **n** Colour profile of the double staining of SHIP2 and Aß42 shows partial association of SHIP2 staining with amyloid plaque (green and red, respectively)

The alteration of SHIP2 solubility led us wonder whether SHIP2 protein localization could be altered in AD brains. We analysed SHIP2 immunolabelling in human brain tissues using anti-SHIP2 (3E6) antibody, well-characterized for specific immunostaining of SHIP2 [11, 20, 26, 31, 32, 63, 73]. Indeed, it has been previously shown that this antibody specifically labels SHIP2 in MCF-7 breast cancer cells but not in the cells deficient for *INPPL1* by CRISPR/Cas9 technology [75] and thus was considered as specific for immunostaining. Stellate SHIP2 positive structures were observed in the hippocampus of AD and control non-demented brains (Fig. 3a–b). The size and intensity of SHIP2 staining were, however, generally increased in AD brains compared to control non-demented cases in the affected brain areas such as hippocampus and temporal cortex. The 3E6-positive immunolabelling was significantly increased and their average size was larger in AD brains compared to control non-demented brains in CA1-2 region of the hippocampus (Fig. 3c–d). Furthermore, SHIP2-immunolabelling showed a significant correlation with 6E10-positive Aß staining (Fig. 3e–g) and AT8-positive phosphotau staining (Fig. 3h–j).

A double immunofluorescence staining confirmed that SHIP2-positive structures were associated with amyloid plaques showing a partial co-labelling (Fig. 3k–n). These data suggest that increased SHIP2-immunolabelling was associated with amyloid pathology in AD brains.

**Fig. 4:**
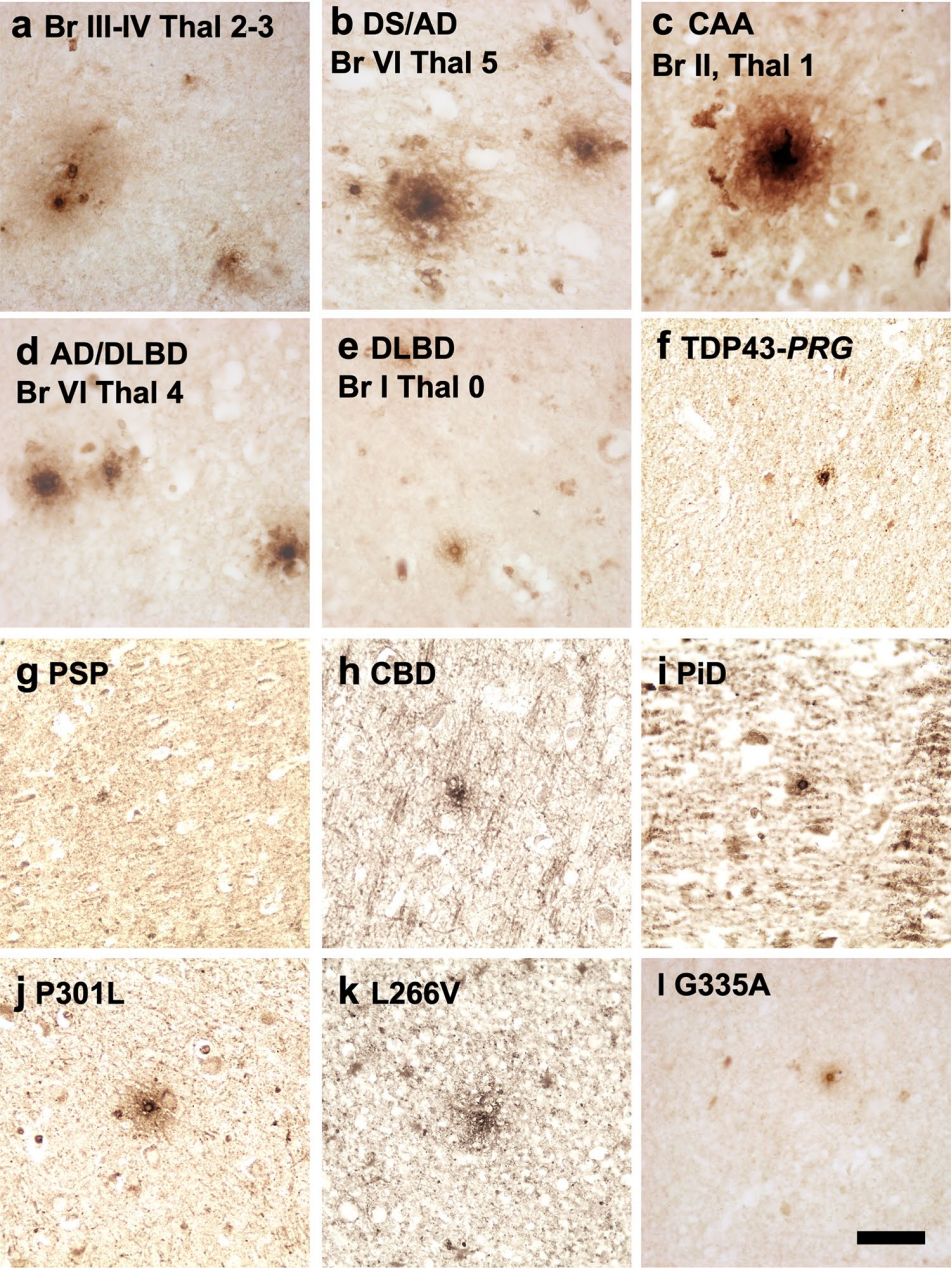
SHIP2 immunoreactivity is increased in the presence of amyloid pathology in the human brain of related neurodegenerative diseases. **a-l** Representative photos of immunostaining of SHIP2 in CA1-2 area of a case of Braak III-IV, Thal 2–3 (**a**), DS/AD (Braak VI, Thal 5) (**b**), CAA (Braak II, Thal 1) (**c**), LBD/AD (Braak VI, Thal 4) (**d**), DLBD without AD lesion (Braak I, Thal 0) (**e**), FTD-U with TDP-43 accumulation due to *PRG* mutation (**f**), PSP (**g**), CBD (**h**) Pick disease (**i**) and FTD-tau with *MAPT* mutation of P301L (**j**), L266V (**k**) and G335A (**l**). There was a global correlation between the levels of amyloid load and SHIP2. Primary tau pathology such as *MAPT-*G335A did not lead to an increased SHIP2 immunoreactivity. CA1-2 area of hippocampus was analysed for **a**-**f**, **i**-**l**, striatum was analysed for PSP cases and temporal cortex was analysed for CBD cases. *Scale bar* 20 μm

To further explore the relationship between the increased SHIP2 labelling and amyloid pathology, we subsequently extended our analyses in the affected brain areas of related neurodegenerative diseases (Fig. 4). SHIP2-immunoreactive structures were increased in the presence of amyloid plaques in pre-AD brains of Braak III-IV (Fig. 4a). Strong immunoreactivity of SHIP2 was observed in a DS/AD case exhibiting severe AD lesions (Fig. 4b), CAA (Fig. 4c) and a combined case of diffuse Lewy body disease with AD (AD/DLBD) (Fig. 4d). There was, however, no clear increase of SHIP2-immunolabelling in the absence of amyloid pathology such as DLBD without AD pathologies, FTD with TDP-43 positive inclusions (Fig. 4e–f) or primary tauopathies such as PSP, CBD, PiD or FTD-tau with *MAPT* mutation at P301L [81], L266V [50] or G335A [5] (Fig. 4j–l). Taken together, these data support the hypothesis that amyloid pathology precedes and leads to increased immunolabelling of SHIP2. Accumulation of other neuropathological proteins such as alpha-synuclein, TDP-43 or tau did not clearly lead to such increase of SHIP2 immunolabelling in the affected brain area.

### Increased SHIP2 labelling is partially associated with amyloid pathology in 5xFAD mouse brains

**Fig. 5:**
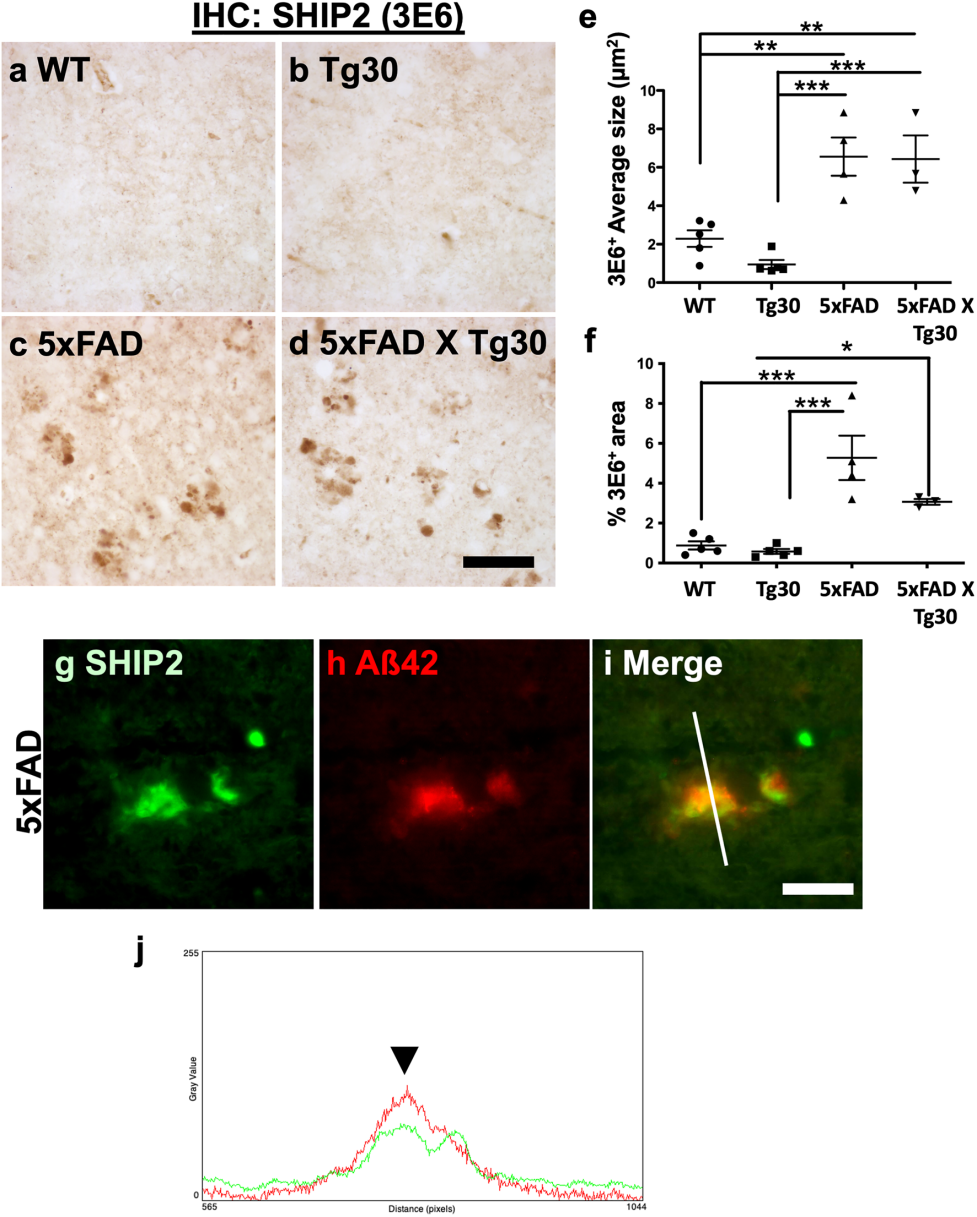
Increased SHIP2 immunoreactivity is associated with amyloid but not with tau pathology. **a-d** Representative images of SHIP2 immunolabelling in the brain stem of 10-month-old male WT (**a**), Tg30 (**b**), 5xFAD (**c**) and 5xFAD X Tg30 (**d**) mice. There was a stronger SHIP2 labelling in 5xFAD and 5xFAD X Tg30 mouse brains. **e–f** Thresholding measurement of the average size (**e**) and area (**f**) immunostained by SHIP2 in the brain stem of WT, Tg30, 5xFAD and 5xFAD X Tg30 mice. The average size and percentage of the area occupied by 3E6 staining were increased in the presence of amyloid pathology but not of tau pathology. *p < 0.05 and **p < 0.01 by *p < 0.05, **p < 0.01 and *** < 0.001 by one-way ANOVA with Tukey post-hoc test. (WT: *n* = 5, Tg30: *n* = 5, 5xFAD: *n* = 4, 5xFAD X Tg30: *n* = 3). **g-i** A double immunostaining for SHIP2 (**g**, *green*) and Aß42 (**h**, *red*) suggested that SHIP2 immunoreactivity was detected in the brain stem of 5xFAD mouse brains. Merged image is shown in **i**. **j** Color profile of the double staining of SHIP2 and Aß42 shows partial association of SHIP2 staining with amyloid plaque (green and red, respectively). *Scale bars* 20 μm.

To recapitulate our observations as in human post-mortem tissues, Tg30 and 5xFAD transgenic mouse models of AD were analysed by immunostaining for SHIP2. Tg30 mice overexpress human double mutant tau [56, 77] while 5xFAD overexpress human mutant APP and PS1 [67]. 5xFAD X Tg30 line was generated by crossing 5xFAD and Tg30 mice and shows exacerbated tauopathy compared to Tg30 but alleviated amyloid pathology than 5xFAD [42]. In line with the observation of the human post-mortem brain tissue of FTD-tau with *MAPT* mutation (Fig. 4j–l), there was no significant difference observed in SHIP2 (3E6) immunolabelling in the brain of Tg30 mice overexpressing human double mutant tau compared to wild-type mice (Fig. 5a–b). Nevertheless, there was a significant increase in the average size and areas of SHIP2-positive structures in 5xFAD and 5xFAD X Tg30 mouse brains (Fig. 5c–f). These data clearly provide evidence of amyloid pathology that triggers increased SHIP2 immunolabelling in the transgenic mouse brains. As observed in human AD brain sections, SHIP2-immunoreactive structures were associated with amyloid plaques in 5xFAD mouse brain by double immunofluorescence staining (Fig. 5g–j). In conclusion, we have observed a clear association between amyloid pathology and increased SHIP2-immunoreactivity. The presence of amyloid pathology triggered an increased SHIP2 immunostaining in transgenic models of amyloid pathology, as observed in human AD brains.

### Loss of function of Ship2 does not cause AD lesions in mouse brains of Ship2^Δ/Δ^

To decipher how the loss of function of SHIP2 may influence AD pathogenesis, we analysed the brain of Ship2^Δ/Δ^ mice that express catalytically inactive truncated Ship2 protein [29]. Immunohistochemistry for anti-Aß, anti-APP, anti-GFAP and anti-Iba1 showed that there were no detectable AD-like lesions observed in Ship2^Δ/Δ^ mouse brains at 6–9 months of age (Supplementary Fig. 2). Analyses showed no obvious tau pathology detected in Ship2^Δ/Δ^ mouse brains by immunostaining using anti-total tau, anti-pTau (PHF-1 and AT8), anti-tau in pathological conformation (MC1) antibodies or by Gallyas silver staining (Supplementary Fig. 3). These data suggest that loss of function of SHIP2 (i.e. catalytic activity) does not trigger detectable AD-lesions in mouse brains.

### SHIP2 is highly expressed in dystrophic neurites and astrocytes associated with amyloid plaques

**Fig. 6:**
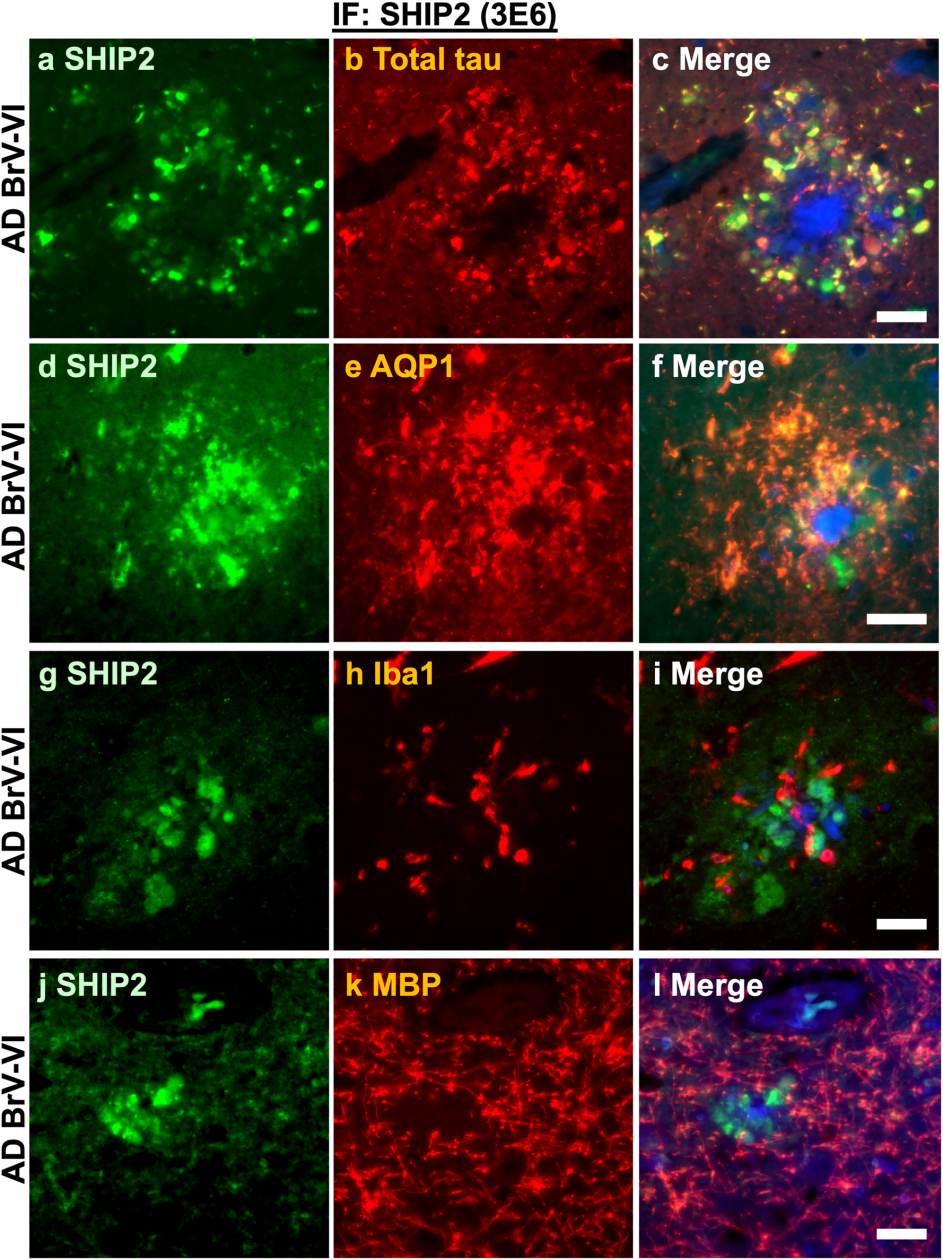
SHIP2 immunoreactive structures were co-stained with plaque-associated tau and astrocytes in AD brains. **a-c** SHIP2 (**a**, green) and tau (**b**, red) were partially co-stained in the AD hippocampus. **d-f** SHIP2 (**d**) and astrocyte marker AQP1 (**e**) were partially co-labelled. **g-i** SHIP2 (**g**) and microglial marker Iba1 (**h**) were not clearly co-labelled. **j-l** SHIP2 (**j**) and oligodendrocyte marker MBP (**k**) were not clearly co-labelled. Merged images show DAPI counterstaining in blue (**c**, **f**, **i**, **l**). *Scale bar* 20 μm

To determine the cell types that accumulate SHIP2 in association with amyloid plaques, double immunofluorescence staining was carried out using anti-SHIP2 antibody and cell specific markers in post-mortem human AD brain sections (Fig. 6). DAPI staining detected cellular nuclei and β-sheet core of compact amyloid plaques (Fig. 6c, f, I, l) [61, 62]. There was a partial co-labelling of plaque-associated dystrophic neurites stained by both anti-SHIP2 (3E6) and anti-tau antibodies (Fig. 6a–c). SHIP2 immunoreactivity was also strongly detected in AQP1-positive astrocytes around amyloid plaques (Fig. 6d–f). On the contrary, SHIP2 immunoreactivity was not clearly detected in Iba1-positive microglia (Fig. 6g–i) nor in MBP-positive oligodendrocytes (Fig. 6j–l). These data suggest that Aß-induced SHIP2 upregulation occurs predominantly in neuronal processes and in astrocytes associated with amyloid plaques.

### SHIP2 overexpression accelerates the formation of FRET positive tau inclusion in HEK tau RD P301L FRET biosensor cells

**Fig. 7:**
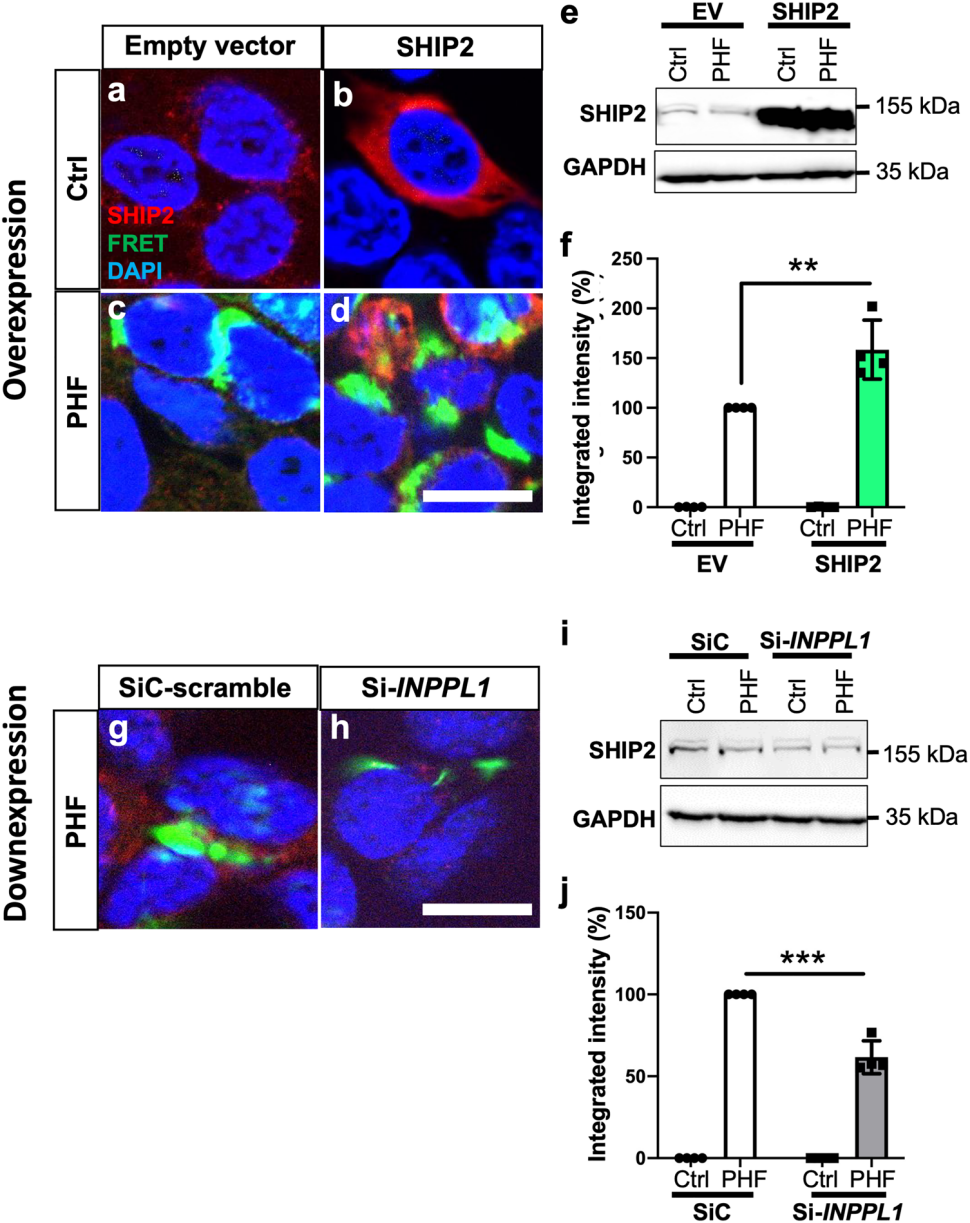
SHIP2 overexpression led to increased FRET signal while siRNA-mediated SHIP2 downexpression led to decreased FRET signal in Tau RD P301S FRET biosensor cells transduced with AD-PHF. **a-d**. Representative image of tau RD P301S FRET biosensor cells fixed 48 h after transduction of AD-PHF and plasmid, immunostained for SHIP2 and counterstained with DAPI. Co-transduction of sarkosyl insoluble fraction from a control case with an empty vector (**a**) or a plasmid encoding SHIP2 (**b**) did not lead to a FRET positive tau inclusion. Transducing AD-PHF (sarkosyl insoluble fraction from an AD brain) led to FRET-positive tau inclusion in this model (**c**, **d**). The cells were co-transduced with AD-PHF and either empty vector (**c**) or SHIP2 plasmid (**d**). **e–f.** SHIP2 overexpression was verified by WB (**e**). There was a significant increase in FRET positive signal when AD-PHF and SHIP2 were co-transduced (**f**). **g-j** Tau RD P301S FRET biosensor cells were co-transduced with AD-PHF and either with a control scramble siRNA (SiC) (**g**) or siRNA targeting *INPPL1* (**h**). There was a significant decrease of SHIP2 expression when siRNA-INPPL1 was transduced (**i**). INPPL1 downexpression led to a decreased FRET signal (**j**). **f**, **j**. 10,000 cells per experiment were analysed by FRET flow cytometry and the results were shown as integrated mean fluorescence intensity. Four independent experiments were carried out for overexpression and downexpression of SHIP2, respectively. ** < 0.01 and *** < 0.001 by two-way ANOVA. *Scale bar* 20 μm

We and others have reported a significant acceleration of tau pathology spreading in the presence of amyloid pathology in the brain of transgenic model for amyloid pathology [41, 85]. The local upregulation of SHIP2 in plaque-associated dystrophic neurites and astrocytes led us wonder whether SHIP2 may accelerate tau pathology and interconnect Aß and tau in AD brains. To decipher the potential role of SHIP2 on tau pathology, we analysed the effect of overexpression of SHIP2 on FRET signal of tau-tau interaction in tau RD P301L FRET biosensor cells [25, 43] (Fig. 7a–f). The biosensor cells were co-transduced with sarkosyl-insoluble fraction from control non-demented brain or from AD case that contains AD-PHF, together with either an empty vector or a plasmid encoding SHIP2. Neither transducing sarkosyl insoluble fraction from a non-demented control brain nor overexpression of SHIP2 led to FRET signal (Fig. 7a–b). By transducing PHF prepared from AD brains, more than 25% of cells showed aggregates, displaying puncta and reticular FRET-positive inclusions (Fig. 7c–d). Co-transduction of AD-PHF and SHIP2 plasmid led to a significant increase of SHIP2 expression (Fig. 7e) and to an approximately 50% increase of the integrated mean fluorescence intensity of FRET signal compared to co-transduction of AD-PHF and control empty vector (Fig. 7f). Conversely, co-transduction of AD-PHF and siRNA targeting *INPPL1* depleting SHIP2 protein led to an approximately 40% decrease of the integrated mean fluorescence intensity of FRET signal in tau RD P301L FRET biosensor cells (Fig. 7g–j). Taken together, these data suggest a potential dose-dependent effect of SHIP2 to accelerate tau–tau interaction in this model of HEK cells.

### rs11235459 in INPPL1 locus is associated with reduced INPPL1 expression and with decreased CSF pTau levels

**Fig. 8:**
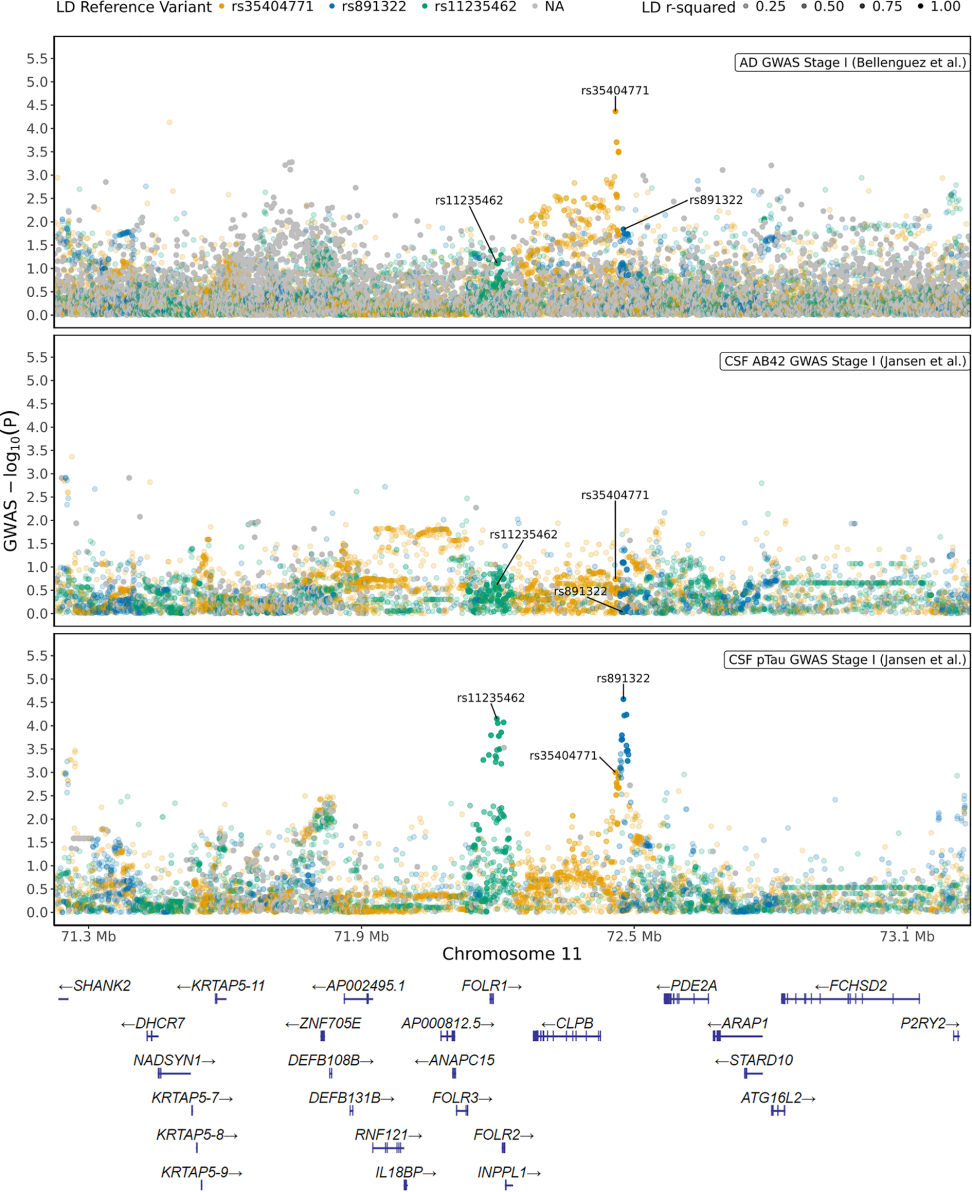
*INPPL1* locus regional plots of AD GWAS, CSF Aß42 GWAS, and CSF pTau GWAS. The association of GWAS variants within 1 Mb of *INPPL1* gene coordinates (hg38 chr11:71,224,767–73,239,147) with AD risk (upper panel, from Bellenguez et al. [13]), with CSF Aß42 (middle panel, from Jansen et al. [46]) and with CSF pTau (lower panel, from Jansen et al. [46]). The top-associated variants for AD risk (rs35404771) and for CSF pTau (rs891322 and rs11235462) independent association signal peaks are labelled on the figure, where their proxy LD patterns are shown in yellow, blue, and green respectively. The colours with higher opacity are indicative for higher LD *r*^*2*^ values. Missing variants in the LD reference panel (1 KG non-Finnish European samples, *n* = 404) are shown in grey. y axis, − log_10_ GWAS *p*; x axis, hg38 genomic position on chromosome 11

**Fig. 9:**
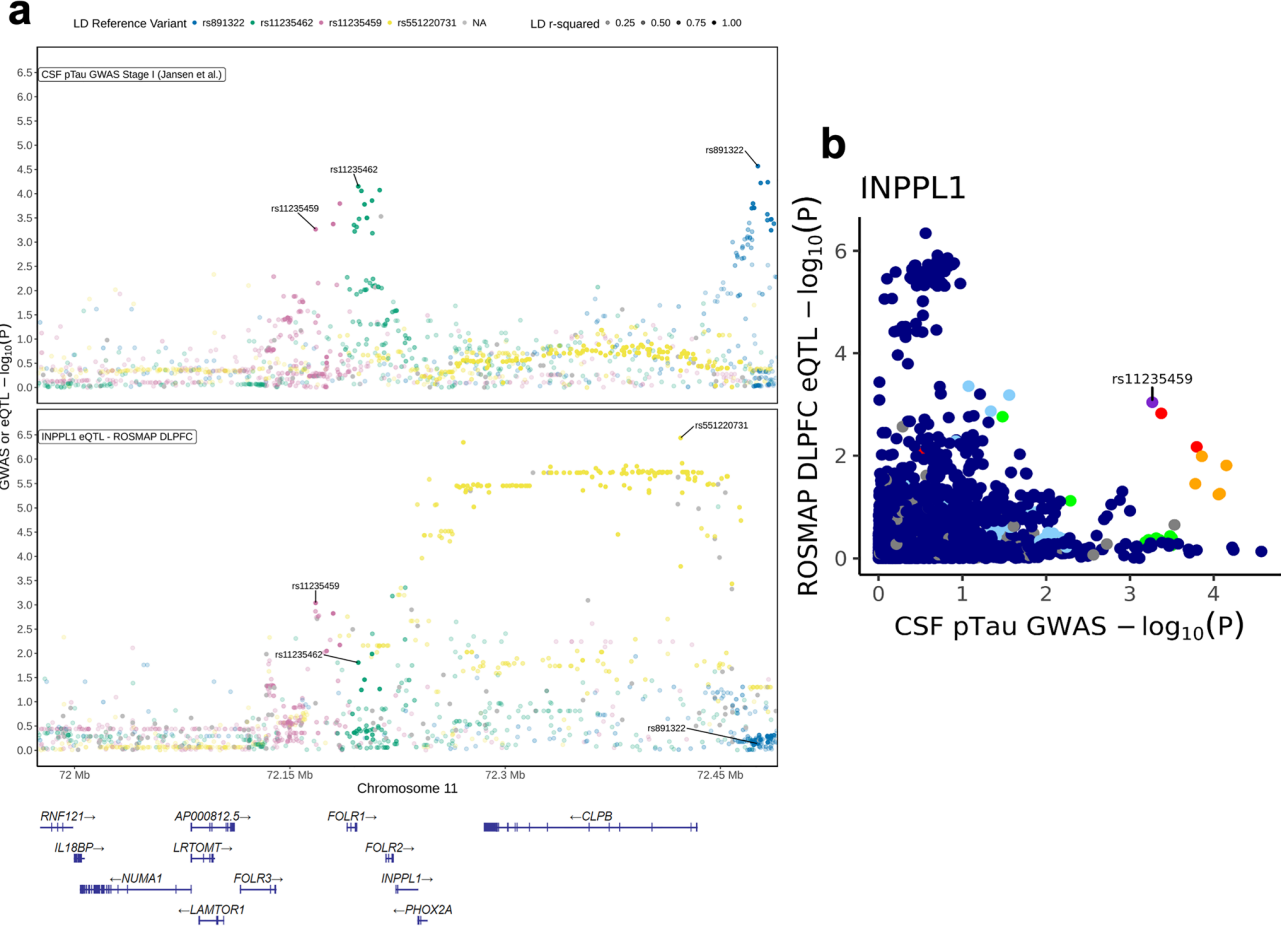
Regulation of *INPPL1* gene expression by the CSF-pTau-associated variants within *INPPL1* locus. **a** The association of GWAS and eQTL variants within 250 kb of *INPPL1* gene coordinates (hg38 chr11:71,974,767–72,489,147) with CSF pTau (upper panel, from [46]) and with *INPPL1* gene expression in brain (lower panel, ROSMAP DLPFC). The top-associated variants for CSF pTau (rs891322 and rs11235462), for *INPPL1* eQTLs (rs551220731) and for both (rs11235459) independent association signal peaks are labelled on the figure, where their proxy LD patterns are shown in blue, green, yellow, and pink respectively. The colours with higher opacity are indicative for higher LD *r*^*2*^ values. Missing variants in the LD reference panel (1 KG non-Finnish European samples, *n* = 404) are shown in grey. y axis, − log_10_ GWAS *p*; x axis, hg38 genomic position on chromosome 11. **b** GWAS-eQTL comparison plot for correlation between ROSMAP DLPFC eQTLs for *INPPL1* gene expression and CSF pTau GWAS. rs11235459 is labelled on the plot, and its LD with other variants are displayed in a colour scale as shown in the legend. Missing variants in the LD reference panel (1 KG non-Finnish European samples, *n* = 404) are shown in grey. y axis, − log_10_ eQTL *p*; x axis, − log_10_ GWAS *p*

Although *EGFR* was recently identified as a novel GWAS hit for LOAD [13], the implication of *INPPL1* genetic variants in relation to AD risk and AD-related biomarker traits remained largely unknown. We, therefore, queried genetic association analyses on *INPPL1*. While there were no suggestive (*p* < 1 × 10^–5^) variants within 1 Mb of *INPPL1* gene coordinates in the stage I GWAS of AD risk [13] and of CSF Aß_42_ and pTau biomarkers [46] (Fig. 8), we observed three subthreshold association signal peaks for AD risk and CSF pTau levels in proximity of *INPPL1*, tagged by three single nucleotide polymorphisms (SNPs). The top-associated SNP for AD risk in the locus was rs35404711 (*p* = 4.3 × 10^–5^, OR = 0.96 [95% CI 0.94–0.98]), while the top-associated SNPs of the two independent peaks (LD *r*^2^ = 0.1) in the CSF pTau GWAS were rs891322 (*p* = 2.7 × 10^–5^, beta = −0.1 [95% CI −0.06 to −0.15]) and rs11235462 (*p* = 7.1 × 10^–5^, beta = −0.09 [95% CI −0.05 to −0.14]). We next investigated whether these potential associations are eQTLs for regulation of *INPPL1* expression in brain, using ROSMAP DLPFC eQTL catalogue (*n* = 560) as a reference (Fig. 9). Colocalization analyses did not show a large overlap between *INPPL1* brain eQTL and CSF pTau GWAS signals in the 1 Mb extended *INPPL1* locus (PP4 = 3.4%) and no strong associations of the top associated *INPPL1* eQTL rs551220731 (not tested in CSF pTau GWAS) and its proxy variants were observed in the CSF pTau GWAS (Fig. 9a). However, we detected an eQTL signal tagged by rs11235459 which was nominally significant in both CSF pTau GWAS (*p* = 5.4 × 10^–4^, OR = 0.93 [95% CI 0.89–0.97]) and *INPPL1* ROSMAP DLPFC eQTL catalogue (*p* = 9.1 × 10^–4^, beta = -0.06 [95% CI −0.02 to −0.09]) (Fig. 9b). The most significant eQTL effect of rs11235459 was observed for *INPPL1* in the 1 Mb extended locus, where rs11235459 was positioned 56 kb upstream of the transcription start site of *INPPL1* and had a minor allele frequency (MAF) of 14.4%. We identified that the minor allele of rs11235459 (C allele) was associated with decreased *INPPL1* gene expression in brain and was also associated with decreased CSF pTau levels.

Finally, by analysing the LD among the five variants of interest, we found that rs11235459 is in a significant LD with rs11235462 (*r*^*2*^ = 0.74) (Supplementary Fig. 4).

We next queried the results for *INPPL1* in the large-scale RVAS for AD risk [44], for AD-relevant biomarker traits [51], and other phenotypic traits [49]. While there was no evidence (*p* > 0.05) of increased burden of rare protein-altering (missense or predicted loss-of-function [pLoF]) mutations with AD risk [44], we detected two nominal associations (*p* < 0.05) with AD-related biomarker traits [51] in cohort-specific analyses in the cohorts of the European Medical Information Framework AD Multimodal Biomarker Discovery (EMIF-AD MBD) and the Alzheimer's Disease Neuroimaging Initiative (ADNI). We observed that the rare missense mutations in *INPPL1* were associated with decreased average cortical thickness in AD signature brain regions (*p* = 0.02) in the EMIF-AD MBD cohort and were associated with increased CSF neurofilament light chain (NfL) levels (*p* = 0.04) in the ADNI cohort [51].

## Discussion

In this study, we assessed the alteration of gene expression, protein level and protein solubility of SHIP2 in AD brains. Upon analysis we found there was a significant upregulation of gene expression of *INPPL1* and its upstream interactor, *EGFR* in the AD brain in the Mayo TCX cohort. In our sample cohorts, we observed an increase in protein levels of SHIP2 and EGFR in the RIPA-insoluble fraction of AD brains despite the significant decrease of SHIP2 in the RIPA-soluble fraction. By immunohistochemistry, we provided evidence that SHIP2 is closely associated with amyloid pathology and that SHIP2 immunoreactivity is upregulated in the presence of amyloid pathology. The increased SHIP2 immunoreactivity was remarkably observed in plaque-associated dystrophic neurites and astrocytes of AD brains. By analysing related neurodegenerative diseases for SHIP2 labelling, we provided clear evidence that amyloid pathology is the key driving factor that leads to the increase in SHIP2 immunoreactivity, but not other neuropathological aggregates such as alpha-synuclein, TDP-43 or tau. Notably, our study in transgenic mouse models suggest that amyloid pathology, but not tau pathology, are necessary to trigger local increase of SHIP2 immunoreactivity around amyloid plaques. A possible consequence of the increase in SHIP2 immunoreactivity could be an upregulation in PI(3,4)P_2_ followed by an activation of RhoA a master regulator of actin-based cytoskeletal dynamics [53]. Local increase of SHIP2 triggered by amyloid pathology may accelerate tau seeding process, as observed in HEK FRET tau biosensor cell assays where SHIP2 overexpression led to increased FRET signal induced by AD-PHF. Our genetic analyses also uncover, for the first time, the potential implication of INPPL1 variants on its expression and CSF pTau level. Our study is in agreement with the previous reports on SHIP2 transcript upregulation in correlation with cognitive deficits and AD lesions in AD and aged brains [65] and on the potential role of SHIP2 as a link of Aβ neurotoxicity to tau pathology in AD [48].

Interestingly, a recent study reports a striking similarity in solubility changes in AD brains between SHIP2 and SHIP1. SHIP1 (encoded by *INPP5D*) is a paralog of SHIP2 and one of the most significant GWAS hits for LOAD [6, 13, 52]. SHIP1 and SHIP2 are differentially expressed: while our double immunofluorescence staining suggested SHIP2 is highly detected in astrocytes (also described in [65]), SHIP1 is predominantly expressed in microglia [22, 84]. It has been recently shown that SHIP1 is depleted in the most soluble fraction of post-mortem AD brain lysates and that SHIP1 immunolabelling is upregulated in AD brain tissues, notably in plaque-associated microglia [22]. Changes in the subcellular localization of SHIP1 and/or SHIP2 may have two consequences: it could affect its phosphatase activity being no more in contact with PI containing membranes, but it could also affect its adaptor function as a docking protein interacting with a cluster of distinct protein interactome in AD [60].

We observed a discrepancy between *INPPL1* and *EGFR* mRNA expression and their protein levels in late Braak stages. Although mRNA expression of *INPPL1* and *EGFR* was increased in AD, the protein level of SHIP2 and EGFR was not changed in total fractions of AD brains. Previous studies have shown that protein levels are generally more conserved than RNA levels in non-proliferating tissues potentially through mechanisms implied in the regulation of protein translation, protein stability or protein degradation [69, 86]. Modifications of the latter mechanisms might explain discrepancy between gene expression and their protein levels of SHIP2 and EGFR in AD.

The protein level of SHIP2 and EGFR was significantly increased in the RIPA-insoluble fraction of AD brain lysate, though their accumulation was quite moderate compared to a strong accumulation of pTau [10] or Aβ [87]. The mechanism through which SHIP2 and EGFR were enriched in the RIPA-insoluble fraction in AD brains remain elusive. The translocation of SHIP2 from soluble to insoluble fraction may be partially due to post-translational modifications regulating protein–protein interaction [28], protein solubility [21] or protein stability [54]. The enrichment of SHIP2 in RIPA-insoluble fraction may also be related to an increased interaction of SHIP2 with insoluble proteins such as cytoskeletal proteins [38] or large protein aggregates such as PHF-tau or amyloid filaments.

Further analyses on post-translational modifications of SHIP2 are necessary to decipher the mechanisms of solubility change of SHIP2 observed in AD brains. SHIP2 is a part of the interactome of the EGFR and is phosphorylated upon EGF activation [70]. SHIP2 can be phosphorylated on more than 20 sites and its phosphorylation regulates subcellular localization and SHIP2 protein stability [30]. SHIP2 can also be ubiquitinylated for its degradation [89]. While abnormal phosphorylation of SHIP2 has been reported in breast cancers [60, 74], it remains largely elusive whether phosphorylation of SHIP2 is altered in AD brains. Since SHIP2 phosphorylation should affect its subcellular localization and solubility, one potential mechanism is that SHIP2 may be trapped in the RIPA-insoluble fraction containing high load of cytoskeletal proteins such as filamin, vinexin, intersectin, RhoA and possibly many adhesome molecules, which have been reported as protein partners of SHIP2 [74]. The protein binding partners of SHIP2 may be altered during the progression of AD. Indeed, both cytoskeletal and adhesion proteins play crucial roles in the formation and the maintenance of synapses as well as regulation of synaptic plasticity [57]. Further analyses on the pathological alterations of SHIP2-protein partners may provide new insights into Alzheimer mechanisms.

A query of the summary statistics of an AD CSF biomarker GWAS revealed nominal associations between genetic variants within *INPPL1* locus and pTau levels in the CSF [46], in addition to being associated with *INPPL1* gene expression in the brain. One limitation for this observation was the relatively limited GWAS sample size due to the complexity of CSF sample collection. In fact, while these association signals were among top 100 significant loci in EADB CSF pTau GWAS stage I results [46], they did not reach the stringent genome-wide significant threshold, and they were slightly above the threshold of *p* < 1 × 10^−5^ which was the criteria for seeking replication in an additional sample of near 5000 additional CSF samples, limiting our ability to further assess these potential associations in a larger cohort. Further larger-scale studies and meta-analyses in various cohorts may reinforce our findings in the future. Nevertheless, together with the association of *INPPL1* variants and CSF pTau levels that we report in this study, our results on FRET-based tau-tau interaction assays in HEK cells are the first evidence that the level of SHIP2 expression affects the tau-tau interaction.

SHIP2 is also implicated in insulin signalling. The single nucleotide polymorphisms of *INPPL1* contribute to the genetic susceptibility to type 2 diabetes and metabolic syndromes [45, 47]. Pharmacological inhibition of SHIP2 has been shown to reduce memory deficits in transgenic mouse models of type2 diabetes [79] and wild-type mice injected with Aß42 [48]. Our study suggests that amyloid pathology triggers a significant accumulation of SHIP2 in plaque-associated dystrophic neurites and astrocytes. Our observations support the hypothesis that EGFR activation of SHIP2 signalling is a sensibilizing factor that is activated in the presence of Aß pathology and accelerates tau pathology in AD brains. Such modifications of SHIP2 could play a role in pathological alterations of PIs, cellular homeostasis in AD brains and could provide new insights for therapeutic approaches targeted to this devastating disease [4]. Furthermore, alterations of SHIP2 localization and solubility may contribute to development of the identification of novel and interesting biomarkers for amyloid pathology and AD.

### Supplementary Information

Below is the link to the electronic supplementary material.

Supplementary file1 (PDF 1787 KB)
