# Supplementary material for: Alteration of gene expression and protein solubility of the PI 5-phosphatase SHIP2 are correlated with Alzheimer’s disease pathology progression
Source: Acta Neuropathol. 2024 Jun 4;147(1):94. doi: 10.1007/s00401-024-02745-7 (PMC11150309; doi:10.1007/s00401-024-02745-7)
Supplement: Supplementary file 1 — Supplementary file1 (PDF 1787 KB) [file 401_2024_2745_MOESM1_ESM.pdf]

Supplementary materials

Alteration of gene expression and protein solubility of the PI 5-phosphatase SHIP2 are correlated with Alzheimer's disease pathology progression

Kunie Ando[1*], Fahri Küçükali[2,3*], Emilie Doeraene[1*], Siranjeevi Nagaraj[1], Eugenia Maria Antonelli[1], May Thazin Htut[1], Zehra Yilmaz[4], Andreea-Claudia Kosa[1], Lidia Lopez-Guitierrez[1], Quintanilla Sanchez Carolina[1], Emmanuel Aydin[1], Ana Raquel Ramos[5], Salwa Mansour[4], Sabrina Turbant[6,7], Brain Bank NeuroCEB Neuropathology Network[6,7], Stéphane Schurmans[8], Kristel Sleegers[2,3], Christophe Erneux[5], Jean-Pierre Brion[1], Karelle Leroy[1]

[1] Alzheimer and other tauopathies research group, ULB Center for Diabetes Research, Medical Faculty, Université Libre de Bruxelles, ULB Neuroscience Institute, 808 route de Lennik, B-1070 Brussels, Belgium.

[2]Complex Genetics of Alzheimer's Disease group, VIB Center for Molecular Neurology, VIB Antwerp, Belgium

[3]Department of Biomedical Sciences, University of Antwerp, Antwerp, Belgium.

[4]Laboratory of Histology, Neuroanatomy and Neuropathology, Faculty of Medicine, Université Libre de Bruxelles, ULB Neuroscience Institute, 808 route de Lennik, B-1070 Brussels, Belgium.

[5]Institute of Interdisciplinary Research in Human and Molecular Biology, Campus Erasme, Université Libre de Bruxelles, 1070 Brussels, Belgium.

[6] Biobanque Neuro-CEB, Hôpital de la Pitié-Salpétrière, Paris, France

[7] Plateforme de Ressources Biologiques (PRB), Hôpital de la Pitié-Salpétrière, Paris, France

[8]Laboratory of Functional Genetics, GIGA Research Centre, University of Liège, Liège, Belgium.

***Authors equally contributed**

Keywords: Alzheimer's disease, SHIP2, INPPL1, EGFR, tau, amyloid ß, GWAS, CSP pTau.

4 Supplementary Figures

1 Supplementary table

Abbreviated title: SHIP2 and AD

Send correspondence to: Dr Kunie Ando and Prof. Karelle Leroy

[1] Alzheimer and other tauopathies research group, ULB Center for Diabetes Research, Medical Faculty, Université Libre de Bruxelles, ULB Neuroscience Institute, 808 route de Lennik, B-1070 Brussels, Belgium.

E-mail: Kunie.Ando@ulb.be and Karelle.Leroy@ulb.be

Abbreviations: SHIP2, SH2 (Src homology 2)-domain-containing inositol phosphatase

Supplementary Figure 1

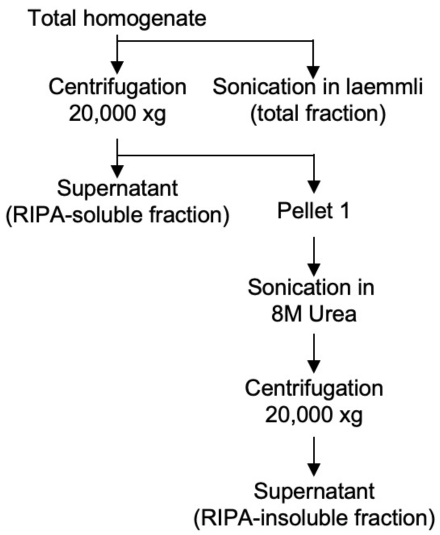

Supplementary Figure 1

Summary of the fractionation protocol to obtain total, RIPA-soluble and RIPA-insoluble fractions. Human T1 isocortex grey matter was fractionated into total, RIPA-soluble and RIPA-insoluble fractions and was analysed by western blotting in this study.

Supplementary Figure 2

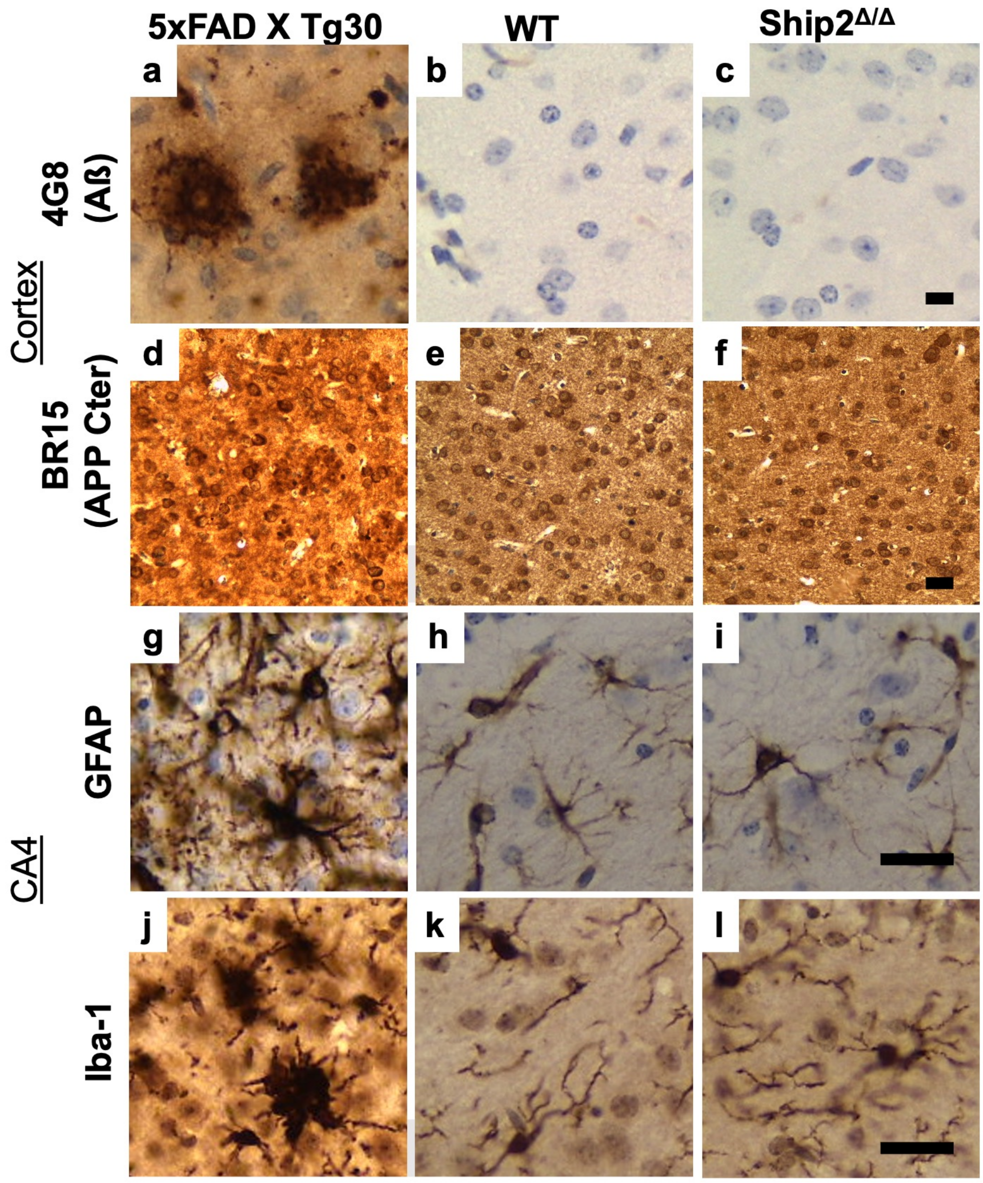

Supplementary Figure 2

Lack of Ship2 catalytic activity did not cause amyloid pathology or gliosis in Ship2$^{\Delta/\Delta}$ mouse brains

Ship2$^{\Delta/\Delta}$ mouse brains showed neither detectable amyloid pathology (**a-c**) nor abnormalities in APP localization (**d-f**) in the cortex. There was no clear astrogliosis (**g-i**) or microgliosis j-l) detected in 6-9 month-old Ship2$^{\Delta/\Delta}$ mouse brains (**c, f, i, l**) compared to wild-type littermates (**b, e, h, k**). 10 month-old male 5xFAD X Tg30 mouse brain was used as a positive control for amyloid pathology and gliosis (**a, d, g, j**). Scale bars 20 µm.

Supplementary Figure 3

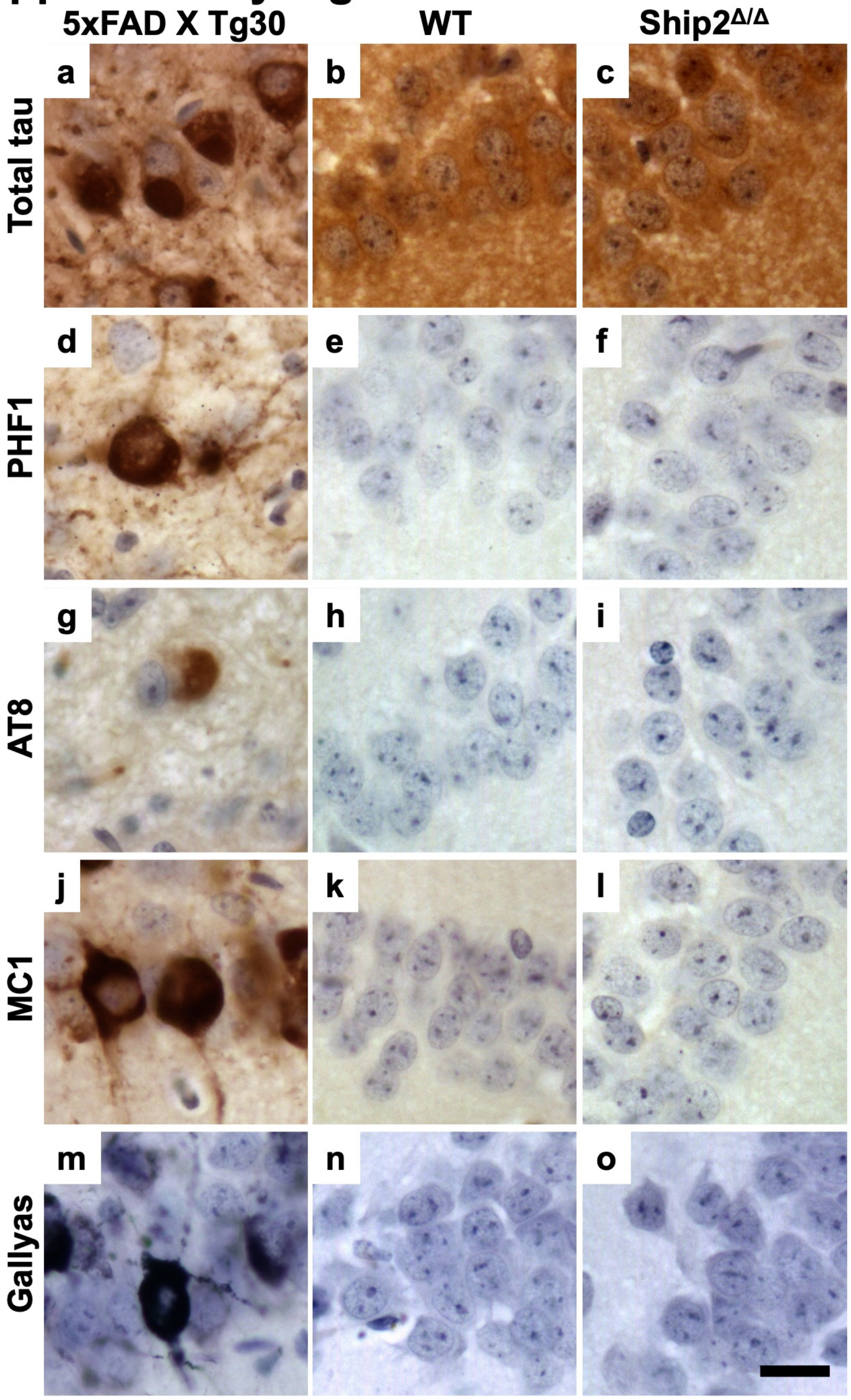

Supplementary Figure 3

Lack of Ship2 catalytic domain did not cause tau lesions in Ship2$^{\Delta/\Delta}$ mouse brains

Ship2$^{\Delta/\Delta}$ mouse brains showed no detectable tau pathology such as somatodendritic tau accumulation (total tau staining in **a-c**), phosphorylation (PHF1 in **d-f**, AT8 in **g-I**), conformation (MC1 in **j-l**) and aggregation (Gallyas staining, **m-o**) of tau in Ship2$^{\Delta/\Delta}$ mouse brains (**c, f, i, l, o**) of 6-9 months old compared to age-matched wild-type littermates (**b, e, h, k, n**). CA1-2 of the hippocampus was analysed. 10 month-old male 5xFAD X Tg30 mouse brain was used as a positive control for tau pathology (**a, d, g, j, m**). Scale bars 20 µm.

Supplementary Figure 4

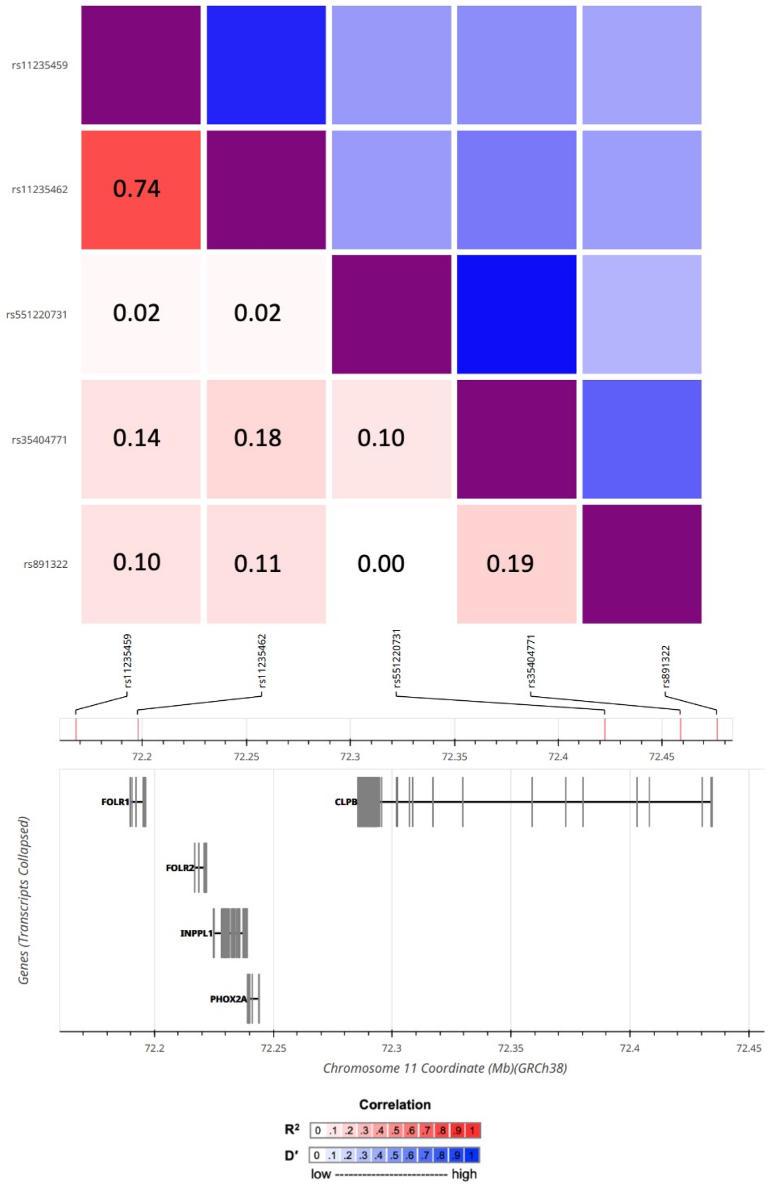

Supplementary Figure 4

LD matrix plot for LD patterns between five variants of interest for GWAS and eQTL analyses

Upper panel shows an LD matrix between five variants of interest for GWAS and eQTL analyses, where boxes for the intersection of these variants are colored based on r^2 values (lower matrix) and D prime (D') values (upper matrix), where higher opacity is indicative for higher r^2 and D' values. Lower panel of the plot shows the collapsed gene models in the locus and the genomic positions for these five variants of interest, where x axis is showing GRCh38 genomic position on chromosome 11. LD reference was based on 1KG non-Finnish European samples (*n*=404). The plot was adapted from “LDmatrix” tool of LDlink portal [2].

Supplementary Table 1

Case #	Clinical diagnostics	Braak	Thal	Sex	Age	PMD	ApoE	Analyses
No. 1	Control	0	0	M	69	6	E2/E3	WB
No. 2	Control	II	0	M	79	NA	E2/E3	WB
No. 3	Control	0	0	F	60	28	E3/E3	WB
No. 4	Control	0	NA	F	86	NA	NA	WB
No. 5	Control	II	0	M	73	10	E3/E3	WB
No. 6	Control	0	1	M	84	13.5	E3/E3	WB
No. 7	Control	I	0	F	92	NA	NA	WB
No. 8	Control	0	NA	M	72	24	E3/E3	WB
No. 9	Control	0-I	NA	M	81	16,5	E3/E3	WB, IHC
No. 10	Control	I	NA	F	89	35	E2/E4	WB
No. 11	Control	0-I	NA	F	71	7	E3/E4	WB
No. 12	Control	I-II	0	M	67	24	E3/E3	WB
No. 13	Control	0-I	NA	M	73	9.5	E3/E4	WB
No. 14	Control	II	1	M	82	63	NA	WB
No. 15	Control	NA	NA	M	71	NA	E3/E3	WB
No. 16	Control	NA	NA	M	80	4	NA	WB
No. 17	Control	NA	NA	F	89	12	NA	WB
No. 18	Control	NA	NA	F	80	NA	NA	WB
No. 19	Control	NA	NA	M	66	NA	NA	WB
No. 20	Control	NA	NA	M	85	NA	NA	WB
No. 21	Control	II	3	M	85	10	NA	WB
No. 22	Control	NA	0	F	52	29	NA	WB
No. 23	Control	II	NA	F	83	21	NA	WB
No. 24	Control	0	NA	M	82	28	NA	WB
No. 25	Control	I-II	0	M	67	24	E3/E3	IHC
No. 26	Control	0	0	F	43	NA	NA	IHC
No. 27	Control	II	0	M	80	8	NA	IHC
No. 28	Control	I	0	F	57	24	NA	IHC
No. 29	Control	I	0	M	65	NA	NA	IHC
No. 30	Control	0	0	M	63	NA	NA	IHC
No. 31	Control	1	4	M	69	NA	NA	IHC
No. 32	Control	III	4	F	82	NA	E3/E3	WB
No. 33	Control	III	NA	M	70	31	E3/E3	WB
No. 34	Control	IV	2	F	76	28	E3/E3	WB
No. 35	Control	IV	1	M	84	48	E3/E3	WB
No. 36	Control	III	4	F	77	48	E3/E4	WB

No. 37	Control	III	2	M	71	24	E3/E4	WB
No. 38	Control	III	3	M	69	24	E3/E4	WB
No. 39	AD	VI	4	M	60	37	E3/E3	WB
No. 40	AD	VI	NA	M	67	19	E3/E4	WB
No. 41	AD	VI	NA	M	57	19	E3/E4	WB
No. 42	AD	VI	4	M	79	28	E3/E4	WB
No. 43	AD	VI	NA	M	63	NA	NA	WB
No. 44	AD	VI	NA	M	74	10	E3/E3	WB
No. 45	AD	VI	NA	M	64	3	E3/E3	WB
No. 46	AD	VI	NA	F	89	10	E2/E3	WB
No. 47	AD	VI	NA	F	87	23	E2/E3	WB
No. 48	AD	VI	5	F	86	30	E3/E3	WB
No. 49	AD	VI	NA	M	71	6	E3/E3	WB
No. 50	AD	VI	NA	F	90	32	E2/E4	WB
No. 51	AD	VI	3	M	61	24	E3/E4	WB
No. 52	AD	VI	4	F	79	24	E3/E4	WB
No. 53	AD	VI	5	M	82	25	E3/E4	WB
No. 54	AD	VI	5	F	83	24.5	E3/E4	WB
No. 55	AD	VI	4	M	83	21	E3/E4	WB
No. 56	AD	VI	NA	M	76	9.5	E3/E4	WB
No. 57	AD	VI	4	F	80	24	E3/E4	WB
No. 58	AD	VI	NA	M	66	9.5	NA	WB
No. 59	AD	VI	NA	M	81	20	E2/E3	WB
No. 60	AD	VI	NA	F	82	20.5	E3/E3	WB
No. 61	AD	VI	5	M	84	NA	E3/E3	WB
No. 62	AD	VI	NA	F	91	26	E3/E3	WB
No. 63	AD	VI	5	F	83	24	E3/E3	WB
No. 64	AD	VI	6	M	81	17.5	E3/E3	WB
No. 65	AD	VI	NA	F	63	28	E3/E4	WB
No. 66	AD	VI	5	F	92	60	E3/E4	WB
No. 67	AD	VI	5	M	76	10	E3/E4	WB
No. 68	AD	VI	5	M	73	45	E3/E4	WB
No. 69	AD	VI	4	M	83	34	E3/E4	WB, IHC
No. 70	AD	VI	NA	M	70	6	E4/E4	WB
No. 71	AD	VI	NA	F	81	8	E3/E3	WB, IHC
No. 72	AD	VI	4	F	72	24	E3/E3	WB, IHC
No. 73	AD	VI	4	F	70	45	E3/E4	WB, IHC
No. 74	AD	VI	4	F	60	24	E3/E3	WB, IHC
No. 75	AD	VI	4	F	76	20	E2/E4	WB, IHC
No. 76	AD	VI	2	F	91	5.5	E3/E4	WB, IHC

No. 77	AD	VI	5	F	70	21	E3/E3	WB, IHC
No. 78	AD	VI	2	M	84	7	E3/E3	WB, IHC
No. 79	AD	VI	5	M	65	30	E3/E3	WB, IHC
No. 80	AD	V	NA	F	88	6	E3/E3	WB
No. 81	AD	V	NA	M	78	24	E3/E3	WB
No. 82	AD	V	NA	NA	NA	NA	E3/E3	WB
No. 83	AD	VI	5	F	89	7	E2/E4	WB, IHC
No. 84	AD	VI	5	F	73	22	E4/E4	WB, IHC
No. 85	AD	VI	NA	NA	82	4.5	E3/E4	WB
No. 86	AD	VI	NA	NA	76	10	E3/E3	WB
No. 87	AD	V	NA	M	74	4	E4/E4	WB, IHC
No. 88	FAD *APP* G2149A	VI	5	F	56	NA	E3/E3	WB
No. 89	FAD *PSEN1* R35E, E120D	VI	5	F	49	14.5	E3/E4	WB
No. 90	AD	V-VI	4	M	89	ND	NA	IHC
No. 91	AD	V-VI	4	F	81	ND	NA	IHC
No. 92	AD	V-VI	4	M	82	ND	NA	IHC
No. 93	AD	V-VI	4	M	75	10	E2/E4	IHC
No. 94	AD	V-VI	4	M	63	NA	NA	IHC
No. 95	AD	V - VI	3	M	46	24	NA	IHC
No. 96	DSAD	VI	5	F	57	ND	E3/E3	IHC
No. 97	CAA	II	1	/	/	NA	NA	IHC
No. 98	DLBD+AD	VI	5	M	79	82	E4/E4	IHC
No. 99	DLBD+early AD	III	3	M	61	24	NA	IHC
No. 100	DLBD	I	0	M	63	60	E3/E4	IHC
No. 101	TDP (C9)	II	0	M	49	NA	NA	IHC
No. 102	TDP (GRN)	0	1	M	72	23	NA	IHC
No. 103	PSP	NA	NA	H	73	38	NA	IHC
No. 104	PSP	NA	NA	F	71	14	NA	IHC
No. 105	PSP	NA	NA	H	71	?	NA	IHC
No. 106	PSP	NA	NA	H	71	23	NA	IHC
No. 107	CBD	NA	NA	F	78	28	NA	IHC
No. 108	CBD	NA	NA	H	66	28	NA	IHC
No. 109	Pick disease	NA	NA	H	61	30	NA	IHC
No. 110	Pick disease	NA	NA	H	77	48	NA	IHC
No. 111	MAPT-P301L	NA	0	M	66	30	NA	IHC
No. 112	MAPT-P301L	NA	0	F	65	31	NA	IHC
No. 113	MAPT-G335A	NA	0	M	34	48	NA	IHC
No. 114	MAPT-L266V	NA	0	F	38	15.5	NA	IHC

Table S1. Human cases analysed in this study.

The neuropathological staging of AD patients is determined according to Braak staging for NFT scores [1] and to Thal staging for amyloid plaque scores [3]. CAA: Cerebral Amyloid Angiopathy. AD: Alzheimer Disease. FAD: familial Alzheimer disease. DSAD: Down syndrome with Alzheimer disease. . Diffuse Lewy body disease: DLBD. TDP; frontotemporal dementia with TAR DNA-binding protein 43 (TDP-43) pathology. Progressive supranuclear palsy: PSP. Corticobasal degeneration: CBD. Microtubule associated protein tau: MAPT. PMD: *post-mortem* delay. NA: not available. Frozen tissues of T1 isocortex were analysed by western blotting (WB). Paraffin embedded tissues of T1 isocortex and hippocampus were analysed by immunohistochemistry (IHC). *APOE* genotyping was determined only for the cases with informed consent for genomic analyses.

References

1 Braak H, Braak E (1991) Neuropathological stageing of Alzheimer-related changes. Acta neuropathologica 82: 239-259 Doi 10.1007/BF00308809

2 Machiela MJ, Chanock SJ (2015) LDlink: a web-based application for exploring population-specific haplotype structure and linking correlated alleles of possible functional variants. Bioinformatics 31: 3555-3557 Doi 10.1093/bioinformatics/btv402

3 Thal DR, Rub U, Orantes M, Braak H (2002) Phases of A beta-deposition in the human brain and its relevance for the development of AD. Neurology 58: 1791-1800
